# Histidine-Triad Hydrolases Provide Resistance to Peptide-Nucleotide Antibiotics

**DOI:** 10.1128/mBio.00497-20

**Published:** 2020-04-07

**Authors:** Eldar Yagmurov, Darya Tsibulskaya, Alexey Livenskyi, Marina Serebryakova, Yury I. Wolf, Sergei Borukhov, Konstantin Severinov, Svetlana Dubiley

**Affiliations:** a Center for Life Sciences, Skolkovo Institute of Science and Technology, Skolkovo, Russia; b Institute of Gene Biology, Russian Academy of Science, Moscow, Russia; c Faculty of Bioengineering and Bioinformatics, Lomonosov Moscow State University, Moscow, Russia; d A.N. Belozersky Institute of Physico-Chemical Biology, Lomonosov Moscow State University, Moscow, Russia; e National Center for Biotechnology Information, National Library of Medicine, Bethesda, Maryland, USA; f Department of Cell Biology and Neuroscience, Rowan University School of Osteopathic Medicine, Stratford, New Jersey, USA; g Center for Precision Genome Editing and Genetic Technologies for Biomedicine, Institute of Gene Biology, Russian Academy of Sciences, Moscow, Russia; h Waksman Institute for Microbiology, Piscataway, New Jersey, USA; National Cancer Institute

**Keywords:** HinT, RiPPs, antibiotics, histidine-triad proteins, microcin C, peptide-nucleotides

## Abstract

The Escherichia coli microcin C (McC) and related compounds are potent Trojan horse peptide-nucleotide antibiotics. The peptide part facilitates transport into sensitive cells. Inside the cell, the peptide part is degraded by nonspecific peptidases releasing an aspartamide-adenylate containing a phosphoramide bond. This nonhydrolyzable compound inhibits aspartyl-tRNA synthetase. In addition to the efficient export of McC outside the producing cells, special mechanisms have evolved to avoid self-toxicity caused by the degradation of the peptide part inside the producers. Here, we report that histidine-triad (HIT) hydrolases encoded in biosynthetic clusters of some McC homologs or by standalone genes confer resistance to McC-like compounds by hydrolyzing the phosphoramide bond in toxic aspartamide-adenosine, rendering them inactive.

## Abstract (precis)

Uncovering the mechanisms of resistance is a required step for countering the looming antibiotic resistance crisis. In this communication, we show how universally conserved histidine-triad hydrolases provide resistance to microcin C, a potent inhibitor of bacterial protein synthesis.

## INTRODUCTION

**FIG 1:**
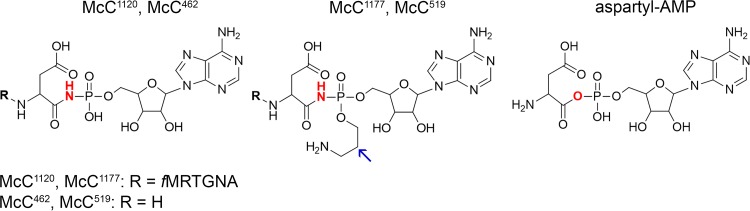
Structures of nonaminopropylated (McC^1120^) and aminopropylated (McC^1177^) E. coli microcin C, processed forms (McC^462^ and McC^519^, respectively), and Asp-AMP, an intermediate of AspRS-catalyzed reaction. The aminopropyl group is indicated by an arrow.

Microcin C (McC) is a ribosomally synthesized posttranslationally modified peptide (RiPP) antibiotic produced by some strains of Escherichia coli. Homologous compounds are encoded by gene clusters in numerous Gram-negative and Gram-positive bacteria (1). McC is produced by E. coli cells harboring a conjugative plasmid containing the *mccABCDEF* cluster (2). The *mccA* gene encodes a seven-amino-acid precursor peptide whose C-terminal residue is modified by the product of the *mccB* gene to yield a peptidyl-adenylate McC^1120^, in which the C-terminal aspartamide is linked to AMP through a nonhydrolyzable *N*-acyl phosphoramidate linkage (Fig. 1) (3). McC^1120^ is further modified by MccD and the N-terminal domain of MccE protein, whose joint action results in a fully matured microcin C, McC^1177^, harboring an aminopropyl decoration on the phosphate moiety (Fig. 1) (4). Both forms of McC are exported from the producing cell by a specialized transporter encoded by the *mccC* gene (5).

McC acts through a Trojan horse mechanism. The peptide part facilitates uptake into the susceptible cell; once inside the cell, the peptide part is proteolytically degraded by aminopeptidases, releasing toxic “processed McC,” a nonhydrolyzable aspartamide-adenylate (Fig. 1), a structural mimic of intermediate of the reaction of aminoacylation of tRNA^Asp^ catalyzed by aspartyl-tRNA synthetase (AspRS) (6, 7). Processed McC competitively inhibits AspRS, bringing protein biosynthesis to a halt (5).

Although most McC is efficiently exported outside the producing cell by the MccC pump, intracellular processing by aminopeptidases should inevitably lead to the accumulation of toxic nonhydrolyzable aspartamide-adenylate and self-intoxication of the producer, since MccC does not export processed McC. Many *mcc*-like clusters acquired additional genes whose products help avoid self-intoxication. In the case of E. coli, the C-terminal domain of MccE, a Gcn5-related *N*-acetyltransferase (GNAT)-type acetyltransferase, acetylates the α-amino group of processed McC, making it unable to bind to AspRS (8). In addition, MccF peptidase cleaves the carboxamide bond between the C-terminal aspartamide and AMP of both intact and processed McC (9).

In this work, we report a novel pathway of McC inactivation by histidine-triad (HIT) superfamily hydrolases encoded in some *mcc*-like biosynthetic clusters or by standalone genes located elsewhere in bacterial genomes. Proteins of the HIT superfamily form two separate functional groups: the first group includes nucleotide hydrolases, represented by HinT (10–13), Fhit (13), APTX (14), and Dcsp (15) enzymes, while the other group includes nucleotide transferases such as GalT (16). The most common members of the HIT superfamily, HinT proteins, were shown to possess phosphoramidase activity (17). We show that bacterial MccH, a product of a gene in an *mcc-*like cluster from Hyalangium minutum, as well as its homologs from Salmonella enterica, Nocardiopsis kunsanensis, and Pseudomonas fluorescens, are phosphoramidases that confer resistance to McC-like compounds by hydrolyzing the toxic aspartamide-adenylate that is produced after intracellular processing of peptidyl-nucleotides.

## RESULTS

### Bioinformatic prediction and experimental validation of an unusual *mcc* operon of Hyalangium minutum.

**FIG 2:**
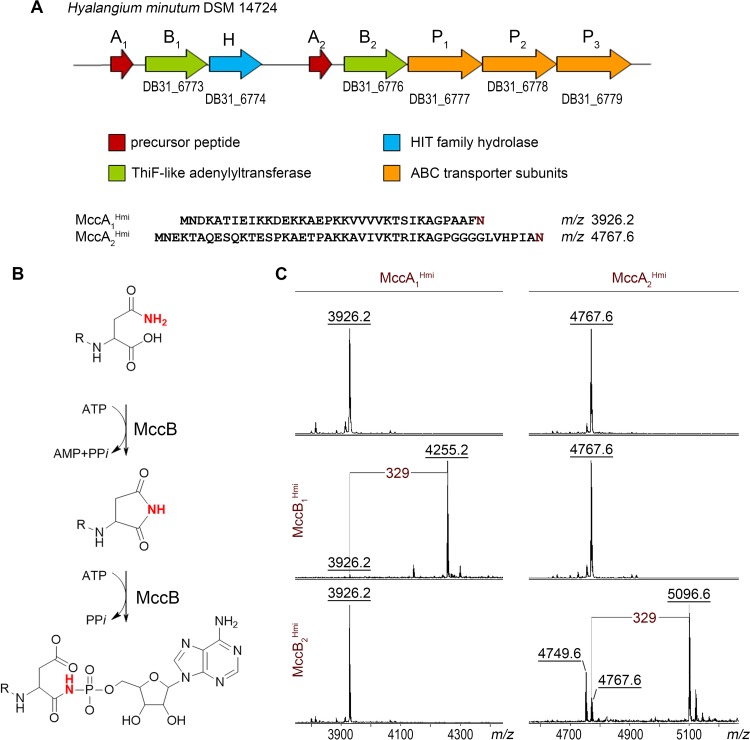
The *mcc*-like operon of *H. minutum* DSM 14724 and its products. (A) Organization of the *mcc*-like gene cluster from *H. minutum* DSM 14724. Genes are represented by colored arrows, with functional predictions corresponding to each color shown in the key beneath. (B) The mechanism of MccB-mediated adenylation of an MccA precursor peptide (3). (C) MALDI-TOF MS spectra of products of *in vitro* reactions between chemically synthesized *H. minutum* MccA_1_ and MccA_2_ precursor peptides and recombinant MccB_1_^Hmi^ and MccB_2_^Hmi^ enzymes in the presence of ATP. Spectra shown at the top are controls (no MccB enzyme added). The mass difference of 329 Da between MccA_1_ (*m/z* 3,926.2) and MccA_2_ (*m/z* 4,767.6) mass ions and the mass ions of the reaction products (*m/z* 4,255.2 and *m/z* 5,096.6, correspondingly) matches the modification with AMP; the MH^+^ ion at *m/z* 4,749.6 present in reaction mixtures containing MccA_2_^Hmi^ and MccB_2_^Hmi^ is a succinimide intermediate of the nucleotidyl transfer reaction.

Bioinformatics analysis reveals a uniquely organized cluster in the genome of the Gram-negative bacterium Hyalangium minutum DSM 14724 that may determine the production of two putative McC-like compounds. The cluster contains two genes coding for putative precursor peptides, MccA_1_ and MccA_2_, two *mccB* genes, encoding THIF-like adenylyl transferases, and three genes whose products likely constitute a complex ABC-type transporter with integrated HlyD-like translocator and C39-like peptidase (18) (Fig. 2A). An additional gene, *mccH*, is located downstream of the *mccB_1_* gene and encodes a protein belonging to a histidine-triad (HIT) superfamily (12).

To validate the predicted *H. minutum mcc*-like cluster, *in vitro* adenylation reactions of synthetic MccA_1_ and MccA_2_ peptides with recombinant MccB_1_ and MccB_2_ adenylyltransferases were performed and products analyzed by matrix-assisted laser desorption ionization–time of flight mass spectrometry (MALDI-TOF MS). As can be seen from Fig. 2B and C, incubation of the 36-amino-acid-long MccA_1_ (MH^+^ at *m/z* 3,926.2) but not the 46-amino-acid-long MccA_2_ (MH^+^ at *m/z* 4,767.6) with MccB_1_ and an equimolar mixture of four nucleotide triphosphates led to the appearance of a mass ion at *m/z* 4,255.2. The 329-Da mass increase corresponds to the adenylated form of the peptide. Conversely, in the presence of MccB_2_, a prominent MH^+^ ion at *m/z* 5,096.6 was observed in reaction mixtures containing MccA_2_, corresponding to its adenylated form. An MH^+^ ion at *m/z* 4,749.6 with 18 mass units less than the original MccA_2_ ion was also detected. It corresponds to an MccB-catalyzed adenylation reaction intermediate, a succinimide derivative of the MccA_2_ peptide. MccA_1_ was not modified by MccB_2_. We conclude that the *H. minutum mcc*-like cluster directs the synthesis of two peptidyl-adenylates, each synthesized from separate precursors by dedicated MccB enzymes.

We next attempted to reconstruct the production of each of the *H. minutum* McC-like compounds in a heterologous E. coli host. Cognate *mccA-mccB* pairs were cloned on one expression plasmid, and the *mccP_1_P_2_P_3_* genes encoding the putative transporter were cloned on a compatible plasmid. Under conditions of induction of plasmid-borne genes, E. coli cells or cellular extracts harboring both plasmid pairs did not inhibit the growth of an McC-sensitive E. coli tester strain, and no mass ions corresponding to *H. minutum* McC-like compounds were detected in cultured medium (see Fig. S1A in the supplemental material). To test if peptidyl-adenylates are synthesized but fail to export from the heterologous host, induced cells were subjected to MALDI-TOF MS. MH^+^ ions at *m/z* 4,255.2 and 5,096.6 corresponding to full-length adenylated MccA_1_ and MccA_2_, respectively, were identified in cells harboring plasmids producing MccA_1_-MccB_1_ and MccA_2_-MccB_2_ pairs. In addition, MH^+^ ions at *m/z* 4,124.2 and 4,965.6, corresponding to adenylated MccA_1_ and MccA_2_ peptides lacking the first methionine residue, were detected. Unmodified full-sized MccA_1_ and MccA_2_ polypeptides (MH^+^ ions at *m/z* 3,926.2 and 4,767.6, respectively) and their derivatives lacking methionine (MH^+^ ions at *m/z* 3,795.2 and 4,636.6) were also observed (Fig. S1B). Thus, two distinct products of the *H. minutum mcc* cluster are produced in the heterologous host but fail to be exported at a detectable level.

10.1128/mBio.00497-20.1 FIG S1 Production of McC_1_^Hmi^ and McC_2_^Hmi^ in a heterologous host. (A). E. coli cells harboring the plasmid-borne *H. minutum mcc* operon do not produce toxic compounds, as follows: *mccA_1_B_1_*, E. coli BL21(DE3) cells harboring pRSF_*mccA_1_B_1_^Hmi^* and pACYC_*mccP_1_P_2_P_3_^Hmi^* plasmids; *mccA_2_B_2_*, BL21(DE3) cells carrying pRSF_*mccA_2_B_2_^Hmi^* and pACYC_*mccP_1_P_2_P_3_^Hmi^* plasmids; and control, E. coli BL21(DE3) cells harboring empty pRSF and pACYC vectors. Cells were induced for 24 h at 30°С and then extracted as described in reference 37. Five microliters of 10× concentrated cell cultures (upper panel) or cellular extracts (lower panel) were deposited on the surface of McC-sensitive E. coli B cells lawn (upper panel). Two microliters of 0.5 μg/ml gentamicin solution was used as a control antibiotic. (B) MALDI-TOF MS analysis of E. coli BL21 cells harboring pRSF_*mccA_2_B_2_^Hmi^* and pACYC_*mccP_1_P_2_P_3_^Hmi^* (top) and pRSF_*mccA_1_B_1_^Hmi^* and pACYC_*mccP_1_P_2_P_3_^Hmi^* plasmids (bottom). At the top spectrum, MH^+^ at *m/z* 5,096.6 corresponding to adenylated MccA_2_^Hmi^, peptide-adenylate lacking N-terminal methionine (MH^+^ at *m/z* 4,965.6), full-length MccA_2_^Hmi^ precursor peptide (MH^+^ at *m/z* 4,767.6), and MccA_2_^Hmi^ lacking N-terminal methionine (MH^+^ at *m/z* 4,636.6) are labeled. MH^+^ ions at *m/z* 3,637.0 and 4,363.5 correspond to E. coli proteins. At the bottom spectrum, ions corresponding to adenylated MccA_1_^Hmi^ (MH^+^ at *m/z* 4,255.2), peptide-adenylate lacking N-terminal methionine (MH^+^ at *m/z* 4,124.2), full-length MccA_1_^Hmi^ precursor peptide (MH^+^ at *m/z* 3,926.2), and MccA_1_^Hmi^ lacking N-terminal methionine (MH^+^ at *m/z* 3,795.2) are labeled. Download FIG S1, PDF file, 0.2 MB. Copyright © 2020 Yagmurov et al. 2020 Yagmurov et al. This content is distributed under the terms of the Creative Commons Attribution 4.0 International license.

### MccH^Hmi^ confers McC immunity when overproduced in E. coli.

**FIG 3:**
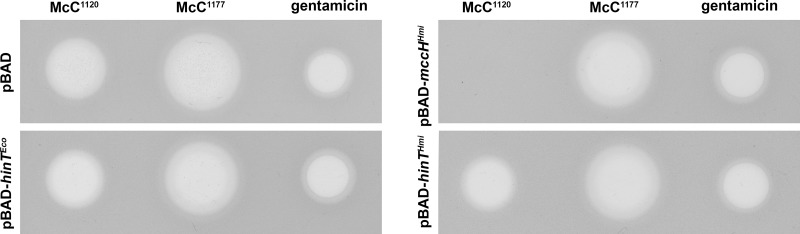
Overproduction of MccH^Hmi^ makes E. coli resistant to McC^1120^ but not to mature E. coli microcin C McC^1177^. Three microliters of 5 μM solutions of McC^1177^, McC^1120^, or 0.5 μg/ml gentamicin used as a control was deposited on lawns of E. coli cells harboring indicated plasmids. The results of overnight growth at 37°C under conditions of the induction of plasmid-borne genes are shown.

We hypothesized that MccH^Hmi^ is a HIT superfamily phosphoramidase that provides *H. minutum* with self-immunity through the inactivation of McC-like compounds. To check this conjecture, we cloned the *mccH^Hmi^* gene in an arabinose-inducible E. coli expression vector. We also created plasmids overproducing HinT^Hmi^, a product of a standalone *H. minutum* gene, and its E. coli homologue HinT^Eco^. Since the final toxic aspartate-adenylate forms of McC produced by *H. minutum* and E. coli should be identical (Fig. 1, McC^462^), we tested the susceptibility of MccH- or HinT-expressing cells to E. coli McC^1120^. Additionally, we used the aminopropylated form of E. coli McC, McC^1177^. In the assay, the drops of solutions of two active forms of E. coli McC were deposited on lawns of E. coli B McC-sensitive cells producing HIT proteins or harboring control empty vector (Fig. 3). The results revealed that the size of the growth inhibition zones around drops of fully mature McC^1177^ solution on lawns of HIT protein-producing cells was the same as that on the control cell lawn. In contrast, E. coli cells overexpressing the *mccH^Hmi^* gene were completely resistant to McC^1120^, an intermediate of the E. coli McC maturation process that does not contain the aminopropyl moiety. The expression of either *hinT^Eco^* or *hinT^Hmi^* had no effect on the size of growth inhibition zones produced by McC^1120^ (Fig. 3). All HIT proteins were produced in comparable amounts, as judged by SDS-PAGE (Fig. S2). We therefore conclude that the MccH^Hmi^ but not HinT proteins tested can provide resistance to externally added toxic peptidyl-adenylate.

10.1128/mBio.00497-20.2 FIG S2 Coomassie-stained SDS polyacrylamide gel showing purified proteins used in the study. Lane L, PageRuler Plus prestained protein ladder; lane 1, MccH^Hmi^; lane 2, MccH^Hmi^ H101N; lane 3, MccH^Hmi^ K103H; lane 4, MccH^Hmi^ F44H; lane 5, HinT^Eco^; lane 6, HinT^Hmi^. Download FIG S2, PDF file, 0.1 MB. Copyright © 2020 Yagmurov et al. 2020 Yagmurov et al. This content is distributed under the terms of the Creative Commons Attribution 4.0 International license.

### MccH^Hmi^ hydrolyzes the phosphoramide bond connecting the aminoacyl and nucleotide moieties of processed McC^1120^.

**FIG 4:**
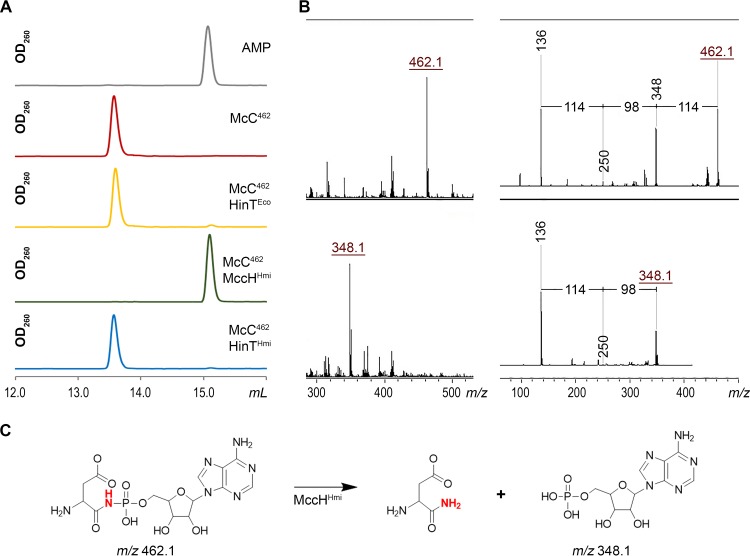
MccH^Hmi^ hydrolyzes aspartamide-adenylate McC^462^. (A) RP HPLC elution profiles of the products of incubation of aspartamide-adenylate McC^462^ with MccH^Hmi^, HinT^Hmi^, and HinT^Eco^. (B) MALDI-TOF MS and MS-MS fragmentation analyses of McC^462^ and the product of its hydrolysis by MccH^Hmi^. For a description of the mass ions, refer to the text. (C) Scheme of an aspartamide-adenylate hydrolysis reaction by MccH^Hmi^.

To determine the mechanism of MccH^Hmi^-mediated resistance to toxic peptidyl-nucleotide, recombinant MccH^Hmi^, HinT^Hmi^, and HinT^Eco^ were purified and incubated with unprocessed McC^1120^ or McC^1177^, and the reaction products were analyzed by reverse-phase high-performance liquid chromatography (RP-HPLC) and MALDI-TOF MS. No changes were observed after a 1-h incubation (data not shown). Since no hydrolytic activity against either form of E. coli McC was detected, we considered whether the processed forms of McC^1120^ and McC^1177^ could be the substrates of MccH^Hmi^. To this end, aspartamide-adenylates with (McC^519^) and without (McC^462^) the aminopropyl group were prepared by *in vitro* processing of McC^1177^ and McC^1120^ (see Materials and Methods). After incubation with MccH^Hmi^, HinT^Hmi^, or HinT^Eco^, samples were analyzed by RP-HPLC and MALDI-TOF MS. Since the expected phosphoramidase activity of MccH^Hmi^ should result in the appearance of AMP or adenosine 5′-phosphoramidate, we used AMP as a marker (Fig. 4A). Upon a 1-h incubation with MccH^Hmi^, McC^462^ was completely converted into a new compound with the same chromatographic mobility as AMP (Fig. 4A). The MH^+^ ion of this compound had *m/z* 348.1, matching that of AMP (Fig. 4B). The MS-MS fragmentation spectra confirmed this assignment (Fig. 4B). None of the enzymes was able to hydrolyze fully processed microcin with aminopropyl decoration (McC^519^) (Fig. S3).

10.1128/mBio.00497-20.3 FIG S3 Aminopropyl decoration of aspartamide-adenylate protects the compound from the phosphoramidase activity of MccH^Hmi^, HinT^Eco^, and HinT^Hmi^. (A) MALDI-TOF MS spectra of McC^519^ incubated without the enzyme (top) and with HinT^Eco^, MccH^Hmi^, and HinT^Hmi^ (bottom). The MH^+^ ion at *m/z* 519.2 corresponds to aminopropylated aspartamide-adenylate. No MH^+^ ion at *m/z* 405.2 corresponding to hydrolyzed McC^519^ is observed. (B) RP-HPLC elution profile of products of incubation of McC^519^, processed aspartamide-adenylate with aminopropyl decoration, without the enzyme and with MccH^Hmi^, HinT^Hmi^, and HinT^Eco^. Download FIG S3, PDF file, 0.1 MB. Copyright © 2020 Yagmurov et al. 2020 Yagmurov et al. This content is distributed under the terms of the Creative Commons Attribution 4.0 International license.

**FIG 5:**
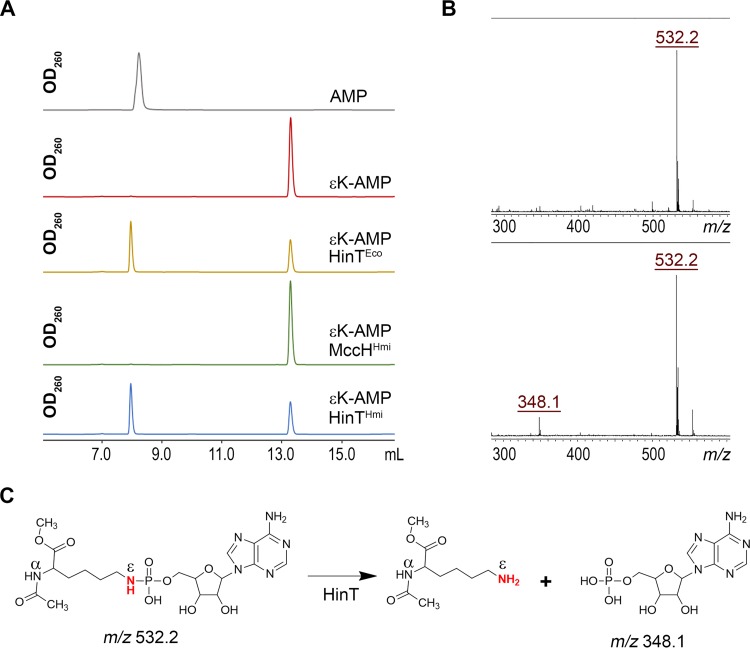
Substrate specificity of MccH^Hmi^, HinT^Hmi^, and HinT^Eco^ phosphoramidases. (A) HPLC elution profiles of products of incubation of εK-AMP with HinT^Eco^, HinT^Hmi^, or MccH^Hmi^. (B) MALDI-TOF MS analysis of the hydrolysis reaction of the εK-AMP by HinT^Hmi^ protein. (C) Scheme of a HinT-mediated hydrolysis reaction of εK-AMP (36).

In the presence of HinT^Eco^ or HinT^Hmi^, McC^462^ remained largely intact, with only trace amounts of AMP formed in the course of the reaction. We therefore decided to assess whether HinT^Hmi^ is an active phosphoramidase using AMP-*N*-ε-(*N*-α-acetyl-lysine methyl ester)-5ʹ-phosphoramidate (εK-AMP), a previously described HinT phosphoramidase model substrate (17). Incubation of εK-AMP with HinT^Hmi^, HinT^Eco^, or MccH^Hmi^, followed by RP-HPLC and MALDI-TOF MS, revealed that both HinT^Hmi^ and HinT^Eco^ hydrolyzed it with the release of AMP, while MccH^Hmi^ did not (Fig. 5A to C). We therefore conclude that the MccH^Hmi^ hydrolase cleaves the P-N bond in aspartamide-adenylate but not in εK-AMP. HinT^Hmi^ and HinT^Eco^ have different specificities, where they hydrolyze εK-AMP well but are largely inactive toward processed McC^1120^ (Fig. 4A and 5A). These results explain why both HinT enzymes failed to provide resistance to McC in the antibiotic susceptibility test under our conditions *in vivo* (Fig. 3).

### MccH homologs are present in diverse bacteria.

**FIG 6:**
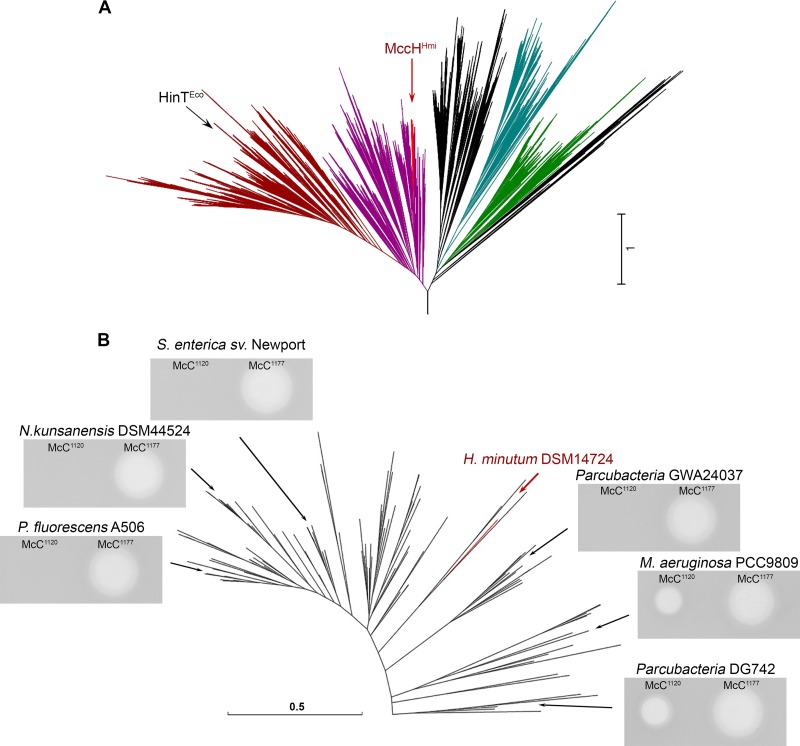
Diversity of McC-specific phosphoramidases. (A) Approximate maximum likelihood (ML) phylogenetic tree of the HIT domain proteins from completely sequenced genomes. Dark magenta and dark red indicate HinT-like proteins (dark red shows the protein kinase C interacting protein-related subgroup), bright red indicates the MccH^Hmi^ clade that is expanded in panel B, cyan indicates GalT, and green indicates FHIT. In black are clades without any clear profile signature. The arrows point to HinT^Eco^ (within the PKCI clade) and MccH^Hmi^. (B) Approximate ML phylogenetic tree of proteins within the MccH^Hmi^ clade and growth inhibition zones formed by McC^1120^ and McC^1177^ on lawns of E. coli B cells transformed with plasmids expressing MccH^Hmi^ or homologs from the indicated positions on the tree. The MccH^Hmi^ branch is highlighted in red. *sv*., serovar.

HIT domain-containing proteins are widespread among prokaryotes (see Materials and Methods for details on domain identification). A phylogenetic tree constructed using available HIT domain sequences revealed that MccH^Hmi^ belongs to a distinct clade, highlighted in red in Fig. 6A (see also Fig. S4). This clade also contains proteins from putative *mcc*-like clusters from *Nocardiopsis*, *Pseudomonas*, and Thermobifida spp., as well as multiple proteins encoded by standalone genes. Genes encoding MccH homologs from the *mcc*-like cluster from Nocardiopsis kunsanensis DSM 44524, as well as standalone genes from Pseudomonas fluorescens A506, Salmonella enterica serovar Newport, Microcystis aeruginosa PCC9809, and Parcubacteria sp. strains GWA24037 and DG742, were cloned into an E. coli expression vector, and the ability of the resulting plasmids to make E. coli cells resistant to the two forms of McC was tested. As can be seen from Fig. 6B, overexpression of most MccH-like genes led to resistance to McC^1120^ but not to McC^1177^. The apparently inactive MccH-like proteins from *M. aeruginosa* strain PCC9809 and *Parcubacteria* sp. strain DG742 are the earliest branching MccH homologs tested, and they may have evolved different substrate specificities.

10.1128/mBio.00497-20.4 FIG S4 Conservation of the amino acid sequence in the protein kinase C interacting protein-related clade of HIT proteins. Shown is a sequence alignment of the HinT clade of HIT proteins (HmiDSM14724a, NCBI RefSeq accession no. WP_044187632.1 of Hyalangium minutum DSM 14724; TteBAA798, GenBank accession no. ACZ41971.1, of *Thermobaculum terrenum* ATCC BAA-798; EcoNCTC9094, NCBI RefSeq accession no. WP_096759427.1 of Escherichia coli NCTC 9094; and SteATCC33386, GenBank accession no. ACZ09064.1 of Sebaldella termitidis ATCC 33386) and the MccH clade of HIT proteins (HmiDSM14724, NCBI RefSeq accession no. WP_044187428.1 of Hyalangium minutum DSM 14724; PflA506, GenBank accession no. AFJ55311.1 of Pseudomonas fluorescens A506; NkuDSM44524, NCBI RefSeq accession no. WP_017574753.1 of Nocardiopsis kunsanensis DSM 44524; SenNewport, ECU0367860.1 of Salmonella enterica subsp. enterica serovar Newport; ParGWA24037, GenBank accession no. KKR61370.1 of *Parcubacteria* sp. strain GW2011_GWA2_40_37; MaePCC9809, GenBank accession no. CCI22782.1 of Microcystis aeruginosa PCC 9809; and ParDG742, GenBank accession no. KPJ57467.1 of *Parcubacteria* sp. strain DG_74_2). Residues conserved in either of the two groups are shown in bold and underlined. The histidine-triad active-site region is indicated by a red-shaded box. Conserved and partially conserved hydrophobic and polar residues forming the nucleotide-binding pocket of HIT proteins are indicated by an asterisk (*). Substitutions of the active-site residues in MccH clade proteins are indicated by ‡. Red boxes mark residues of “inactive” MaePCC9809 and ParDG742 MccH-like proteins, which differ considerably from the MccH consensus. Download FIG S4, PDF file, 0.4 MB. Copyright © 2020 Yagmurov et al. 2020 Yagmurov et al. This content is distributed under the terms of the Creative Commons Attribution 4.0 International license.

### Mutational analysis of MccH active center.

The mechanism of nucleotide phosphoramidate hydrolysis is best studied for the E. coli enzyme, HinT^Eco^ (19), and its human homologue, hHint1 (20, 21). The characteristic feature of the HIT superfamily proteins is a conserved histidine-triad motif, HxHxHxx, where H is a histidine, and x is a hydrophobic residue (12). The three essential catalytic histidines form a network of hydrogen bonds with the substrate that promotes proton transfer from the protonated C-terminal histidine of the triad (H103 in HinT^Eco^ or H102 in HinT^Hmi^) to phosphoramidate unbridged oxygens and amide nitrogen and facilitate nucleophilic attack of the central histidine (HinT^Eco^ H101 or HinT^Hmi^ H100) on the phosphorus atom, resulting in P-N bond hydrolysis (19, 21, 22). Another conserved His residue, H39 in HinT^Eco^ (H38 in HinT^Hmi^), located outside the triad motif closer to the N terminus of HIT enzymes, contributes to catalysis by stimulating the protonation of the third histidine of the triad by stabilizing its cationic state (21). Mutational analysis of human Hint1, a close homologue of Hint^Eco^, revealed that substitutions of conserved histidines equivalent to H39 and H103 in HinT^Eco^ substantially reduce the catalytic activity (22).

**FIG 7:**
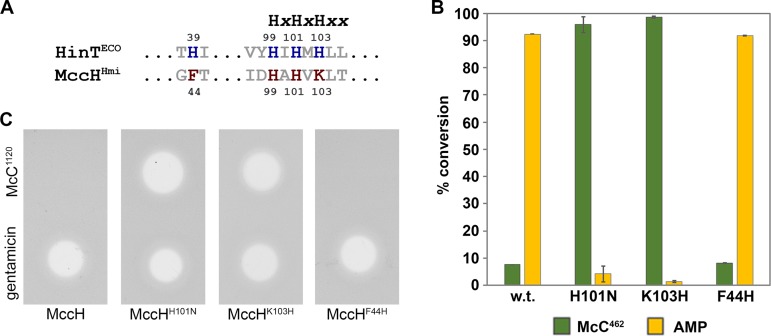
Mutational analysis of active site residues of MccH^Hmi^. (A) Sequence alignment of the conserved HIT motif of HinT^Eco^ and MccH^Hmi^. (B) Phosphoramidase activity of MccH^Hmi^ active-site mutants. *In vitro* reaction mixtures containing aspartamide-adenylate McC^462^ were incubated with wild-type MccH^Hmi^ and the mutant proteins, containing the H101N or K103N substitution, and then analyzed by RP-HPLC. The conversion of McC^462^ was calculated as the percentage of McC^462^ and AMP absorption peak areas that remained after the reaction completion relative to the corresponding peak areas observed without the addition of enzymes. The bars represent the mean ± standard deviation (SD) conversion percentages calculated from three independent measurements. (C) Mutations in the active center of MccH^Hmi^ abolish immunity to McC^1120^. Growth inhibition of E. coli cells harboring pBAD plasmids encoding the indicated proteins. Solutions (5 μM) of McC^1120^ and 0.5 μg/ml gentamicin were deposited on freshly prepared lawns and allowed to grow overnight at 37°C under conditions of induction of plasmid-borne genes.

**FIG 8:**
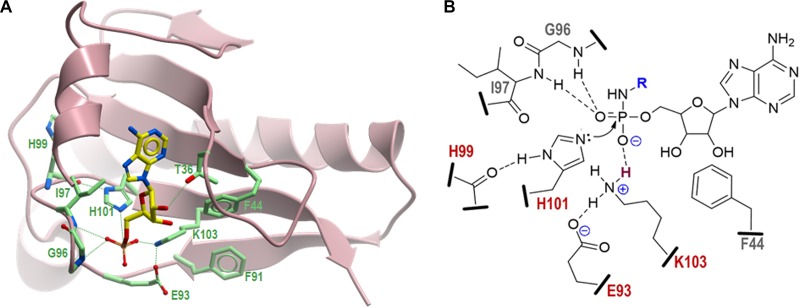
Three-dimensional structural model of MccH^Hmi^ and proposed catalytic mechanism. (A) Model structure of MccH^Hmi^ generated by the SWISS-MODEL homology modeling server (23) using as the template the crystal structures of the HIT-like protein from Mycobacterium paratuberculosis (PDB 3P0T) (24) and human HINT1-AMP complex (PDB 3TW2) (28). Positions of catalytic residues in the MccH^Hmi^ active site in complex with AMP are shown as atom type-colored sticks: N, blue; O, red; P, orange. The C atoms in AMP are in yellow, and the C atoms in the side chains are in light green. (B) Schematic diagram representing the active site of MccH^Hmi^ with the bound substrate, aspartamide-adenylate (the aspartyl moiety is represented by R and shown in blue). Residues of the ExxxxxHxHxKxx motif conserved among all members of the MccH clade that are directly involved in the catalysis are shown in red. Potential hydrogen bonds between peptide side chains, backbone atoms, and phosphate oxygens are indicated by dashed lines. The proton to be transferred from the ε-amino group of K103 to the unbridged oxygen of the phosphoramide is depicted in dark red. Nucleophilic attack of the unprotonated nitrogen of H101 on electrophilic phosphorus atom is shown by a curved arrow.

Interestingly, HIT proteins of the MccH clade contain a modified motif where the third histidine is substituted for lysine (K103 in MccH^Hmi^). Together with this substitution, MccH-like proteins lost the N-terminal conserved histidine (H39 in HintEco), which is replaced by phenylalanine (F44 in MccH^Hmi^) (Fig. 7A and S4). Structural modeling (see below) indicates that F44 makes hydrophobic contacts with the side chain of K103 in MccH^Hmi^ (Fig. 8A). In addition, all members of MccH clade acquired glutamate (E93 in MccH^Hmi^) five residues away from the triad motif. In the structural model of MccH^Hmi^, E93 makes a hydrogen bond (or a salt bridge) with K103, thus playing the same functional role as H39 in HinT^Eco^ (H38 in HinT^Hmi^) (Fig. 8). Since this triple substitution should still allow phosphoramide bond hydrolysis, we speculate that the positively charged lysine occupying position of H103 in MccH^Hmi^ ultimately donates its stationary proton to the nitrogen of the phosphoramide bond (Fig. 8B). To test this conjecture and better understand the origin of MccH-like protein specificity, we prepared two MccH^Hmi^ mutants harboring the single substitutions K103H and F44H. An MccH^Hmi^ K103H-F44H double mutant was also engineered. As a key residue in the triad, H101 in MccH^Hmi^ is supposed to directly participate in catalysis. Therefore, a protein with substitution H101N was prepared and tested for phosphoramidase activity. As expected, the H101N mutant, which served as a control, was catalytically inactive, i.e., no hydrolysis of processed McC^1120^ was detected (Fig. 7B). The K103H substitution had also eliminated the hydrolytic activity of MccH^Hmi^, confirming that a lysine characteristic of MccH-like proteins is essential for the catalytic function. The F44H mutant retained its activity, which is also an expected result. The K103H-F44H double mutant was insoluble, and thus, its enzymatic properties could not be assessed. To confirm the *in vitro* hydrolytic activity of the mutants, the E. coli cells harboring the corresponding plasmids were tested for their susceptibility to McC^1120^. As shown in Fig. 7C, the H101N and K103H substitutions completely abolished immunity to peptidyl-adenylates, while the phenotype of MccH^Hmi^ F44H-expressing cells was indistinguishable from that of the cells producing wild-type MccH^Hmi^.

### Structural model of MccH^Hmi^.

The loss of functional activity by MccH^Hmi^ mutant K103H suggested that this residue, which is specific to MccH clade proteins, is involved in substrate binding and/or catalysis. We also hypothesized that some other active-site residues might spatially constrain the catalytic pocket environment favoring the flexible aliphatic side chain of lysine over a more rigid imidazole ring of histidine. To explore the possible spatial organization of the active center of MccH^Hmi^, we generated its three-dimensional (3D) model using the SWISS-MODEL homology modeling program (23). The resulting model of MccH^Hmi^ (Fig. 8A) is based on a top-ranked Mycobacterium paratuberculosis HIT-like protein structure (PDB 3P0T) (24) with a global model quality estimate (GMQE) quality score of 0.68, indicative of good reliability and accuracy. To reveal the potential interactions in the substrate-binding pocket, the model structure of MccH^Hmi^ was superimposed with the crystal structure of the human histidine-triad nucleotide-binding protein 1 (hHINT1) in complex with AMP (PDB 3TW2) (25).

As expected, the overall structure of MccH^Hmi^ and the spatial organization of its active site are very similar to those of other HinT and HIT-like proteins, with a notable exception of the C-terminal nonconserved 45 amino acids that model differently depending on the homology template used. In the model, MccH^Hmi^ forms a symmetric homodimer with each protein monomer capable of binding and hydrolyzing the substrate (Fig. S5). The nucleoside-binding pocket is formed mostly by conserved hydrophobic residues F11, F12, L15, F34, P37, V46, F38, and I97. The hydroxyl group of T36 makes a hydrogen bond with ribose 2′-OH in AMP, thus contributing to nucleotide recognition. The N atoms of the side chains of catalytic H101 and K103 are positioned (2.5 to 2.7 Å) to make strong hydrogen bonds with the P and unbridged O atoms of the phosphate moiety of AMP, respectively (Fig. 8A). The interatomic distances (2.9 to 3.6 Å) between the peptide backbone amide and carbonyl groups of residues G96, I97, and H99 and their interacting partners (unbridged oxygen and the protonated N atom of H101, respectively) are within a range that is optimal for hydrogen bonding and consistent with the proposed catalytic mechanism (Fig. 8B). Unexpectedly, the carbonyl group of E93, a conserved residue among members of the MccH clade, is in close proximity (2.3 Å) to the protonated N atom of K103, suggesting a strong hydrogen bond or salt bridge that would stabilize the charged state of K103 and facilitate the catalysis. Furthermore, consistent with the results of our mutagenesis experiments (Fig. 7), the bulky hydrophobic side chains of F44 and F91 replacing conserved H39 and I86 in HinT^Eco^ and HinT^Hmi^, about the side chain of K103, stabilize its conformation and direct it toward the phosphate. Thus, the positioning of F44, F91, and E93 in the active center explains the observed preference for a catalytic lysine in MccH instead of histidine in the HinT clade.

10.1128/mBio.00497-20.5 FIG S5 Three-dimensional structural model of MccH^Hmi^ dimer in complex with AMP. The two monomers of MccH are depicted in light-green- and purple-colored ribbon diagrams. Residues of the active site that form the substrate-binding pocket are labeled and shown in a stick representation. Download FIG S5, PDF file, 0.1 MB. Copyright © 2020 Yagmurov et al. 2020 Yagmurov et al. This content is distributed under the terms of the Creative Commons Attribution 4.0 International license.

## DISCUSSION

In this work, we uncover a novel mechanism of immunity to microcin C-like compounds by MccH^Hmi^, a HIT-like phosphoramidase encoded in the *mcc* cluster of *H. minutum*. The cluster produces two separate peptide-adenylates that are analogous to McC^1120^, a toxic maturation intermediate of E. coli McC that lacks the aminopropyl decoration. As of today, the *mcc* operon from *H. minutum* is the only validated operon that produces two McC-like compounds with different peptide parts. Since peptide parts determine the specificity of antibacterial action by allowing selective import into sensitive cells (26), *H. minutum* DSM 14724 may target distinct, nonoverlapping sets of its competitors by the McC-like compounds it produces.

Like other McC-producing organisms, *H. minutum* should experience the buildup of toxic processed products inside the cell, which could lead to the cessation of protein biosynthesis. The MccH^Hmi^ enzyme, the product of the *mcc* operon, alleviates this problem by cleaving the bond between phosphorus and nitrogen in the toxic aspartamide-adenylate that is produced after proteolytic processing of either of the two McC-like compounds encoded by the operon, thus providing self-immunity to the producing cell. MccH^Hmi^ makes cells resistant to the maturation intermediate of E. coli McC that lacks the aminopropyl decoration but not to fully mature McC^1177^. The catalytic mechanism of phosphoramide hydrolysis requires a transient protonation of two unbridged oxygens (21, 22). The presence of an additional aminopropyl group on the phosphate in unprocessed McC^1177^ and processed McC^519^ precludes the proton transfer reaction and renders the phosphorus center inaccessible to nucleophilic attack by the catalytic histidine (HinT^Eco^ H101, HinT^Hmi^ H100, or MccH^Hmi^ H101). Thus, *H. minutum* and E. coli *mcc* operons use different strategies to overcome the self-intoxication of producers. The *H. minutum mcc* operon produces two peptidyl-adenylates without additional modifications, and the processing of both compounds leads to identical toxic aspartamide-adenylate. MccH^Hmi^ hydrolyzes the phosphoramide bond in aspartamide-adenylate with the formation of AMP and aspartamide. The absence of additional genes in the *H. minutum mcc* operon that may be involved in self-immunity suggests that MccH^Hmi^ is sufficient to counter the inhibitory effects caused by the buildup of the toxic product. In E. coli, the MccD/E enzyme complex installs the aminopropyl decoration at the phosphate of peptide-adenylate, which allows the potency of antibacterial action to be increased ∼10-fold by increasing the affinity of the processed compound to its target, AspRS (4). The presence of activity-enhancing decoration renders the MccH^Hmi^ enzyme inactive, necessitating another mechanism to overcome self-intoxication. MccE detoxifies both aminopropylated and nonaminopropylated aspartamide-adenylates by acetylating the amino group of the aspartate (8).

The structural model of MccH^Hmi^ built based on a crystal structure of homologous HIT-like protein from M. paratuberculosis (PDB 3P0T) (24) provides a plausible view on a spatial organization of the active center and offers clues to understanding the enzyme’s substrate specificity. Importantly, the model points to the functional role of the conserved hydrophobic (F44 and F91) and charged (E93) residues in the activation of catalytic K103 for the hydrolysis of aspartamide-adenylate that can be tested experimentally. It is also predicted that residues M95 and W115 of the C-terminal loop of one MccH monomer together with L110 from the adjacent C-terminal loop of the other MccH monomer form a tight aspartamide-binding site which would sterically occlude the binding of bulkier groups, such as the ε-lysine amide of εK-AMP. This view is consistent with the fact that HinT^Eco^ lacking the C-terminal extension present in MccH^Hmi^ was active toward εK-AMP but could not hydrolyze aspartamide-adenylate. It is also supported by previous observations that both deletion and swapping of the C-terminal loop between human HINT1 and HinT^Eco^ strongly affect both the catalytic activity and substrate specificity (19, 27, 28). The proposed model will be validated in our future genetic, biochemical, and structural studies of bacterial MccH and HinT proteins.

Previous studies have shown that bacterial and human HinT proteins exhibit a broad substrate specificity; they can accommodate both purine and pyrimidine nucleotides with various substitutions in the aminoacyl moiety, including d- and l-stereoisomers of tryptophan and sterically hindering *N*-ε-(*N*-α-acetyl-lysine methyl ester)-adenosine phosphoramidates (11, 27). Unlike HinT, the MccH^Hmi^ and its homologues from four diverse bacterial species characterized in this work apparently have evolved much more specialized enzymes that show a clear preference for aspartamide-adenylate (Fig. 4 and 5). Our results suggest that the fully processed McC is a bona fide substrate for MccH. We speculate that MccH-like proteins from *M. aeruginosa* PCC9809 and *Parcubacteria* sp. DG742, which are inactive toward E. coli McC, may recognize yet-unidentified species-specific McC-like compounds that carry different nucleoside or amino acid moieties.

## MATERIALS AND METHODS

### Molecular cloning.

E. coli DH5α was used for cloning. All primers were synthesized by Evrogen (Russia); their sequences are listed in Table S1. The *H. minutum* DSM 14724 or E. coli BW25113 genomic DNA was used as the template for PCR. Phusion DNA polymerase (Thermo Scientific) was used for PCR.

10.1128/mBio.00497-20.7 TABLE S1 Primers used in the study. Download Tabl e S1, PDF file, 0.1 MB. Copyright © 2020 Yagmurov et al. 2020 Yagmurov et al. This content is distributed under the terms of the Creative Commons Attribution 4.0 International license.

For MccB_1_^Hmi^ and MccB_2_^Hmi^ activity analysis, their coding sequences were amplified from the *H. minutum* DSM 14724 genomic DNA. The PCR products were digested with BamHI and SalI restriction endonucleases and inserted under the same sites into the pET22_MBP vector (26) to create an N-terminal fusion protein with a maltose-binding protein (MBP) tag.

For the heterologous *mcc^Hmi^* expression system, a DNA fragment spanning the *mccP_1_^Hmi^*, *mccP_2_^Hmi^*, and *mccP_3_^Hmi^* genes was amplified from genomic DNA, digested with EcoRI and KpnI, and inserted into pACYCDuet-1 vector (Novagen-Millipore, USA) linearized with the same restriction endonucleases. To construct MccA-MccB expression vectors, first, the phosphorylated self-complementary oligonucleotides containing sequences of the MccA_1_^Hmi^ and MccA_2_^Hmi^ open reading frames (ORFs) were inserted into the pRSFDuet-1 vector (Novagen-Millipore, USA) and digested with NcoI and HindIII, resulting in the pRSF-MccA_1_^Hmi^ and pRSF-MccA_2_^Hmi^ vectors, respectively. Then, *mccB_1_^Hmi^* and *mccB_2_^Hmi^* were PCR amplified, digested with NdeI and KpnI, and inserted into the pRSF_*mccA_1_^Hmi^* and pRSF_*mccA_2_^Hmi^* vectors linearized with NdeI and KpnI, resulting in pRSF_*mccA_1_B_1_^Hmi^* and pRSF_*mccA_2_B_2_^Hmi^*, respectively.

To obtain an arabinose-inducible vector for the heterologous expression of HIT proteins, the pBAD/His B vector (Invitrogen-Thermo Fisher, USA) was linearized by PCR with the appropriate primers that contained an introduced ribosomal binding site and SalI and HindIII restriction sites to generate pBAD30_SalRBS. Next, the *mccH^Hmi^*, *hinT^Hmi^*, and *hinT^Eco^* genes were PCR amplified using genomic DNA as the template and the corresponding primers. Genes encoding the homologs of MccH^Hmi^ were purchased as synthetic DNA fragments from IDT, USA. All amplified PCR products and synthetic fragments were digested with SalI and HindIII and inserted between the same sites into the pBAD30 expression vector.

To create vectors for HIT protein fused with C-terminal His_6_ tags for protein purification, the *mccH^Hmi^*, *hinT^Hmi^*, and *hinT^Eco^* genes were PCR amplified, digested with NdeI and XhoI, and inserted into the pET22(b) vector (Novagen-Millipore, USA). The site-directed mutagenesis of *mccH^Hmi^* was carried out using overlap extension PCR (29), with appropriate primers.

### Recombinant protein expression and purification.

Recombinant proteins were produced in E. coli BL21(DE3) transformed with the appropriate plasmid. The cells were grown in 500 ml of TB medium supplemented with ampicillin to an optical density at 600 nm (OD_600_) of ∼0.7 and induced with 0.2 mM isopropyl-β-d-thiogalactopyranoside (IPTG). After induction, the culture was transferred for overnight growth at 18°C and 180 rpm. The cells were harvested by centrifugation at 8,000 × *g* and 4°C for 20 min, resuspended in ice-cold resuspension buffer (20 mM Tris-HCl, 300 mM NaCl, 1 mM dithiothreitol [DTT] [pH 8.0]), supplemented with 1 mM phenylmethylsulfonyl fluoride (PMSF), and disrupted by sonication. For the His-tagged proteins, imidazole was added up to 2 mM. The lysate was cleared by centrifugation at 30,000 × *g* and 4°C for 20 min. The cleared lysate was applied to a preequilibrated column with a tag-binding resin; depending on the tag present, either amylose resin (NEB) or Talon CellThru Co^2+^-chelating resin (TaKaRa-Clontech) was used. The resin was washed with 10 column volumes of resuspension buffer, followed by elution with 5 column volumes of the elution buffer (20 mM Tris-HCl [pH 8.0], 50 mM NaCl, 10% glycerol) supplemented with either 10 mM maltose or 0.5 M imidazole.

### Adenylation of MccA_1_^Hmi^ and MccA_2_^Hmi^.

For validation of the MccA-MccB pairs of the *H. minutum mcc*-like gene cluster, *in vitro* modification of synthetic MccA_1_^Hmi^ (MNDKATIEIKKDEKKAEPKKVVVVKTSIKAGPAAFN) and MccA_2_^Hmi^ (MNEKTAQESQKTESPKAETPAKKAVIVKTRIKAGPGGGGLVHPIAN) (GenScript, USA) peptides using purified MccB_1_^Hmi^ and MccB_2_^Hmi^ reactions was performed. Reaction mixtures contained 50 μM synthetic peptides (either MccA_1_^Hmi^ or MccA_2_^Hmi^), 5 μM recombinant MccB_1_^Hmi^ or MccB_2_^Hmi^, and 2 mM each nucleoside triphosphate (NTP) in the reaction buffer (50 mM Tris-HCl [pH 8.0], 150 mM NaCl, 10 mM MgCl_2_, 5 mM DTT). The reaction was carried out at 30°С for 16 h and then stopped by the addition of 0.1% trifluoroacetic acid (TFA) in water. The products of the reaction were analyzed by MALDI-TOF MS for the presence of adenylated MccA_1_^Hmi^ and MccA_2_^Hmi^.

### McC_1_^Hmi^ and McC_2_^Hmi^ production test.

E. coli BL21(DE3) cells harboring a combination of either pRSF_*mccA_1_B_1_^Hmi^* and pACYC_*mccP_1_P_2_P_3_^Hmi^* or pRSF_*mccA_2_B_2_^Hmi^* and pACYC_*mccP_1_P_2_P_3_^Hmi^* plasmids were grown in 25 ml of 2xYT medium (1.6% Tripton, 1% yeast extract, 0.5% NaCl) at 30°C with constant shaking at 180 rpm. Upon reaching an OD_260_ of ∼0.7, the cells were induced with 0.25 mM IPTG and grown for an additional 20 h at 30°C. After that, the cells were pelleted at 5,000 × *g* and resuspended in 250 μl of M9 medium. Aliquots of 15 μl were deposited on a freshly prepared M9 agar lawn of E. coli strain B harboring the pRSFduet-1, pETduet-1, and pACYCduet-1 vectors. Plates were incubated at 30°C overnight to allow formation of the lawn. The next day, the plates were inspected for the presence of growth inhibition zones. Additionally, the cells and the surrounding agar were analyzed for the presence of McC-like products using mass spectrometry.

### *In vivo* immunity assay.

E. coli strain B was transformed with pBAD_SalRBS vectors containing the *mccH^Hmi^* gene or its homologues. The overnight cultures of the E. coli cells with the appropriate vectors were diluted 1,000-fold in M9 agar medium supplemented with 1% glycerol, 0.02% yeast extract, 10 mM arabinose, and 100 μg/ml ampicillin. The sensitivity of cells carrying plasmid-borne genes encoding HIT-like proteins was measured by applying 5-μl drops of 5 μM McC^1120^ and 5 μM McC^1177^ on the surface of the plate and allowed to dry, and gentamicin (0.5 μg/ml) was used as a control growth inhibitor. Plates were incubated for 16 h at 30°C for a lawn to form. The next day, the growth inhibition zones were analyzed.

### εK-AMP synthesis.

The synthesis of εK-AMP was performed as described elsewhere, with minor modifications (17). To synthesize εK-AMP, 0.12 g (6.25 mmol) of 1-ethyl-3-(3-dimethylaminopropyl)carbodiimide HCl (Sigma-Aldrich) was added to the flask containing 0.98 g (2.7 mmol) of AMP (Sigma-Aldrich), 0.1 g of (0.42 mmol) *N*-α-acetyl-l-lysine methyl ester HCl (Sigma-Aldrich), and 10 ml of ultrapure water, and the pH of the solution was adjusted with triethylamine to a value of 7.5. The mixture was incubated in the shaker at 65°C, with vigorous shaking at 250 rpm for 22 h. After allowing the reaction mix to cool down to room temperature, the mixture was lyophilized, redissolved in 0.1% TFA in water, and applied to a Luna 5-μm C_18_ 100-Å, LC column (250 by 10 mm; Phenomenex). The purification of εK-AMP was performed in a linear gradient (5 to 25%) of acetonitrile. The fractions were analyzed for the presence of εK-AMP by MALDI-TOF mass spectrometry, and fractions containing εK-AMP were subjected to additional chromatographic purification on the same column in a linear gradient of acetonitrile (0 to 25%) in triethylammonium acetate (TEAA) buffer (pH 6.5).

### *In vitro* phosphoramidase activity assay.

To test the phosphoramidase activity of MccH^Hmi^, HinT^Hmi^, HinT^Eco^ enzymes, and their respective variants, the processed forms of E. coli McC^1177^ and McC^1120^ (McC^519^ and McC^462^, respectively) and εK-AMP were used as the substrates. For production, purification, and processing of McC forms, refer to the study by Metlitskaya et al. (6). Fifty micromolar processed McC or εK-AMP was mixed with 5 μM the enzyme in the reaction buffer (20 mM HEPES [pH 7.2], 2.5 mM MgCl_2_, 2.5 mM MnCl_2_). The reaction mixture was incubated at 25°С for 30 min, terminated by the addition of 0.1% TFA, and analyzed for hydrolysis by HPLC and mass spectrometry.

### Reverse-phase HPLC analysis of the products of the *in vitro* reactions.

All biochemical reactions were analyzed on 1220 Infinity II LC system (Agilent), and the peak separation occurred on a Zorbax Eclipse Plus C_18_ 5-μm (4.6 by 250 mm) column (Agilent) in a 0.1 M TEAA buffer system (pH 6.0) in the varying linear gradient of acetonitrile.

The products of McC^462^ hydrolysis reactions were separated in a linear gradient of acetonitrile (0 to 20%) over a period of 15 min. After the incubation of McC^519^ with HIT enzymes, the reaction products were separated in the acetonitrile gradient (0 to 22%) for 15 min. After hydrolysis of εK-AMP by HIT enzymes, the reaction products were analyzed in the linear acetonitrile gradient (5 to 30%) lasting for 15 min. The chromatograms were processed with the use of the ChemStation software (Agilent), and elution profiles were exported in comma-separated values format.

### Mass spectrometry analysis.

One to two microliters of the sample aliquots was mixed with 0.5 μl of matrix mix (Sigma-Aldrich) on a steel target. The mass spectra were recorded on an UltrafleXtreme MALDI-TOF/TOF mass spectrometer (Bruker Daltonics) equipped with a neodymium laser. The molecular MH^+^ ions were measured in reflector mode; the accuracy of the measured results was within 0.1 Da.

### Sequence analysis.

Proteins containing the histidine catalytic triad were identified in 4,621 completely sequenced genomes available in 2016. Profiles belonging to the NCBI CDD (30) superfamily cl00228 were used as PSI-BLAST (31) queries to search the protein sequences encoded in this set. The resulting set of 10,580 proteins was clustered using UCLUST (32) and aligned using MUSCLE (33) (Fig. S6); alignments were iteratively compared to each other using HHSEARCH and aligned using the HHALIGN program (34). The approximate ML tree was reconstructed using the FastTree program (35) with a WAG evolutionary model and gamma-distributed site rates.

10.1128/mBio.00497-20.6 FIG S6 Alignment of cluster consensus sequences of the HIT domain proteins from completely sequenced genomes. The phylogenetic tree, constructed from the multiple alignment of 15,351 HIT sequences (Fig. 6A), was split into subtrees at the average depth of 1.5 from the tree tips, producing 292 clusters of 4+ sequences. Consensus sequences were derived for each cluster from the corresponding subsets of the alignment (positions less than 30% conserved denoted by “x”). Positions corresponding to active-site histidine residues are highlighted in yellow. Consensus sequences for the MccH^Hmi^ (CON.109), HinT^Eco^ (CON.19), and HinT^Hmi^ (CON.21) clades are shown in red font. Download FIG S6, PDF file, 0.3 MB. Copyright © 2020 Yagmurov et al. 2020 Yagmurov et al. This content is distributed under the terms of the Creative Commons Attribution 4.0 International license.
