# Supplementary material for: Histidine-Triad Hydrolases Provide Resistance to Peptide-Nucleotide Antibiotics
Source: mBio. 2020 Apr 7;11(2):e00497-20. doi: 10.1128/mBio.00497-20 (PMC7157772; doi:10.1128/mBio.00497-20)
Supplement: FIG S1 [file mBio.00497-20-sf001.pdf]

SUPPLEMENTARY MATERIALS

Histidine-Triad Hydrolases Provide Resistance to Peptide-Nucleotide Antibiotics

Eldar Yagmurov[1], Darya Tsibulskaya[1,2], Alexey Livenskyi[2,3] Marina Serebryakova[2,4], Yury I. Wolf[5], Sergei Borukhov[6], Konstantin Severinov[1,7,8 *], and Svetlana Dubiley[1,2 *]

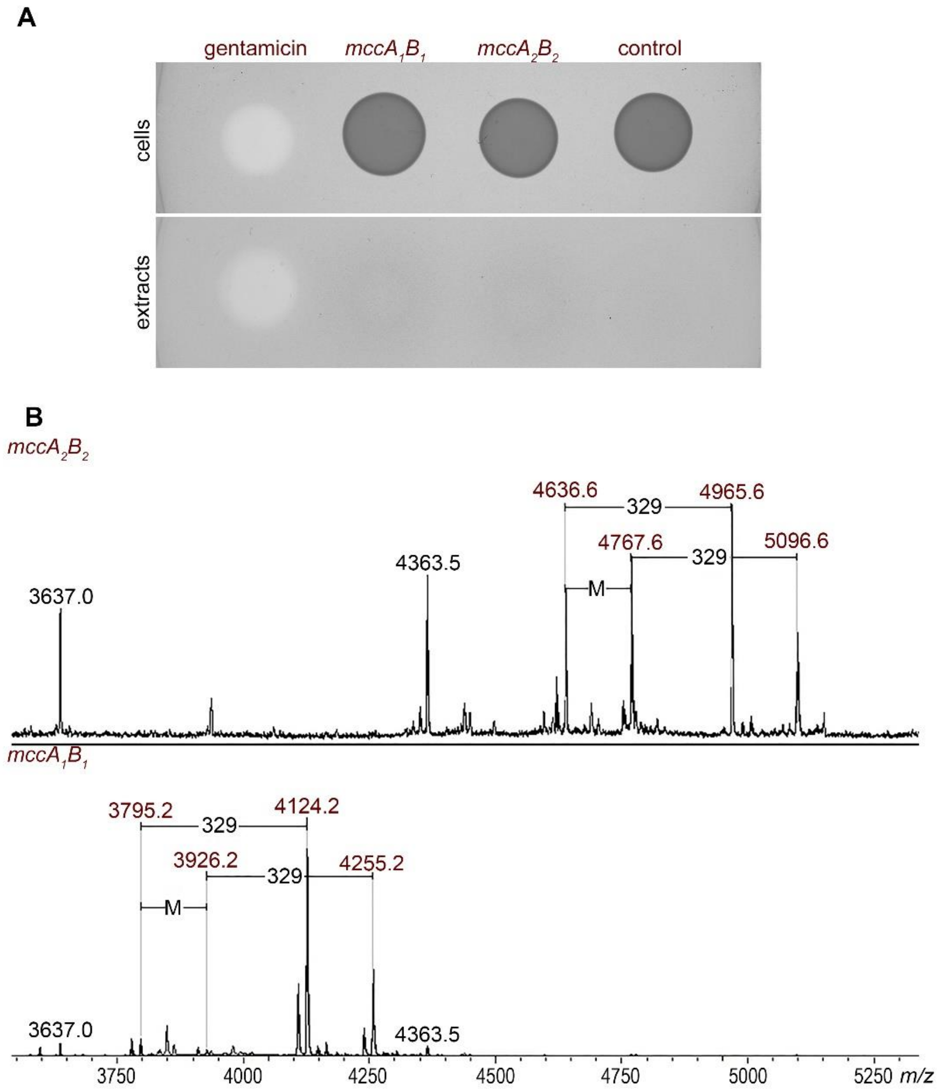

Figure S1. Production of McC$_1^{Hmi}$ and McC$_2^{Hmi}$ in heterologous host.

(A) *E. coli* cells harboring plasmid-borne *H. minutum mcc* operon do not produce toxic compounds: *mccA₁B₁* – *E. coli* BL21(DE3) cells harboring pRSF_$mccA_1B_1^{Hmi}$ and pACYC_$mccP_1P_2P_3^{Hmi}$ plasmids, $mccA_2B_2$ – BL21(DE3) cells carrying pRSF_$mccA_2B_2^{Hmi}$ and

pACYC_*mccP*$_1$*P*$_2$*P*$_3^{Hmi}$ plasmids, control - *E. coli* BL21(DE3) cells harboring empty pRSF and pACYC vectors. Cells were induced for 24 h at 30 °C, then extracted as described in (37). 5 µl of 10-times concentrated cell cultures (upper panel) or cellular extractes (lower panel) were deposited on the surface of McC-sensitive *E. coli* B cells lawn (upper panel). 2 µl of 0.5 µg/mL gentamycin solution was used as a control antibiotic.

(B) MALDI-TOF-MS analysis of *E. coli* BL21 cells harboring pRSF_*mccA*$_2$*B*$_2^{Hmi}$ and pACYC_*mccP*$_1$*P*$_2$*P*$_3^{Hmi}$ (upper panel) and pRSF_*mccA*$_1$*B*$_1^{Hmi}$ and pACYC_*mccP*$_1$*P*$_2$*P*$_3^{Hmi}$ plasmids (lower panel)**.** At the top spectrum, MH^+ at *m/z* 5096.6 corresponding to adenylated $MccA_2^{Hmi}$, peptide-adenylate lacking N-terminal methionine (MH^+ at *m/z* 4965.6), full-length $MccA_2^{Hmi}$ precursor peptide (MH^+ at *m/z* 4767.6), and $MccA_2^{Hmi}$ lacking N-terminal methionine (MH^+ at *m/z* 4636.6) are labeled. MH^+ ions at *m/z* 3637.0 and 4363.5 correspond to *E. coli* proteins. At the bottom spectrum, ions corresponding to adenylated $MccA_1^{Hmi}$ (MH^+ at *m/z* 4255.2), peptide-adenylate lacking N-terminal methionine (MH^+ at *m/z* 4124.2), full-length $MccA_1^{Hmi}$ precursor peptide (MH^+ at *m/z* 3926.2), and $MccA_1^{Hmi}$ lacking N-terminal methionine (MH^+ at *m/z* 3795.2) are labeled.
