# Supplementary material for: Histidine-Triad Hydrolases Provide Resistance to Peptide-Nucleotide Antibiotics
Source: mBio. 2020 Apr 7;11(2):e00497-20. doi: 10.1128/mBio.00497-20 (PMC7157772; doi:10.1128/mBio.00497-20)
Supplement: FIG S2 [file mBio.00497-20-sf002.pdf]

SUPPLEMENTARY MATERIALS

Histidine-Triad Hydrolases Provide Resistance to Peptide-Nucleotide Antibiotics

Eldar Yagmurov[1], Darya Tsibulskaya[1,2], Alexey Livenskyi[2,3] Marina Serebryakova[2,4], Yury I. Wolf[5], Sergei Borukhov[6], Konstantin Severinov[1,7,8 *], and Svetlana Dubiley[1,2 *]

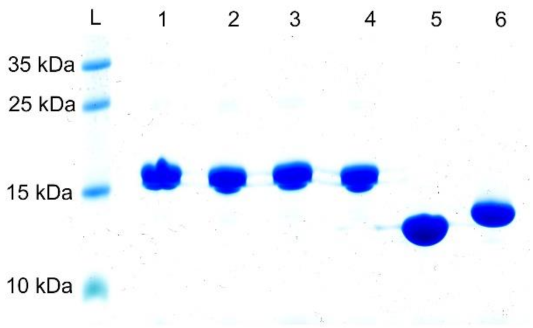

Figure S2. Coomassie-stained SDS polyacrylamide gel showing purified proteins used in the study. L – PageRuler Plus Prestained protein ladder; 1 – MccH^{Hmi}; 2 – MccH^{Hmi} H101N; 3 – MccH^{Hmi} K103H; 4 – MccH^{Hmi} F44H; 5- HinT^{Eco}; 6 - HinT^{Hmi}.
