# Supplementary material for: Histidine-Triad Hydrolases Provide Resistance to Peptide-Nucleotide Antibiotics
Source: mBio. 2020 Apr 7;11(2):e00497-20. doi: 10.1128/mBio.00497-20 (PMC7157772; doi:10.1128/mBio.00497-20)
Supplement: FIG S3 [file mBio.00497-20-sf003.pdf]

SUPPLEMENTARY MATERIALS

Histidine-Triad Hydrolases Provide Resistance to Peptide-Nucleotide Antibiotics

Eldar Yagmurov[1], Darya Tsibulskaya[1,2], Alexey Livenskyi[2,3] Marina Serebryakova[2,4], Yury I. Wolf[5], Sergei Borukhov[6], Konstantin Severinov[1,7,8 *], and Svetlana Dubiley[1,2 *]

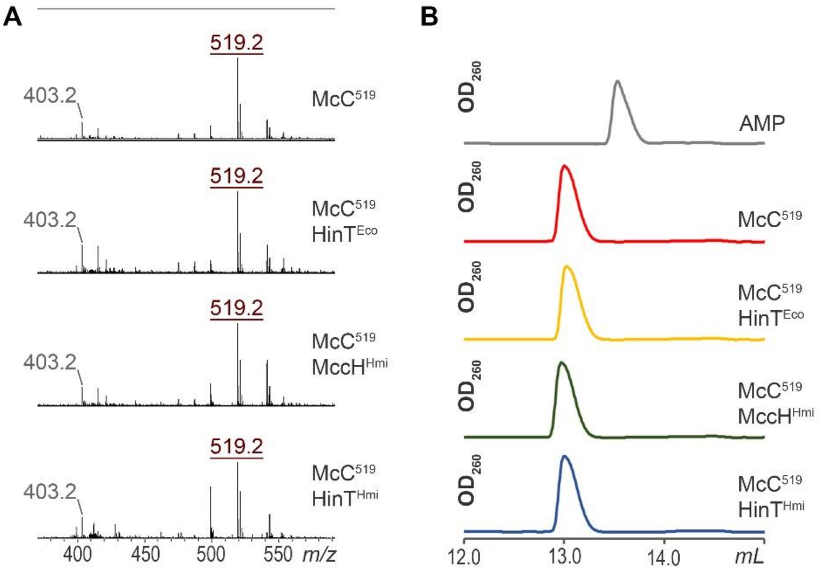

Figure S3. Aminopropyl decoration of aspartamide-adenylate protects the compound from the phosphoramidase activity of MccH^{Hmi}, HinT^{Eco}, and HinT^{Hmi}.

(A) MALDI-TOF-MS spectra of McC^{519} incubated without the enzyme (top panel) and with HinT^{Eco}, MccH^{Hmi}, and HinT^{Hmi} (lower panels). The MH$^+$ ion at *m/z* 519.2 corresponds to aminopropylated aspartamide-adenylate. No MH$^+$ ion at *m/z* 405.2 corresponding to hydrolyzed McC^{519} is observed.

(B) RP-HPLC elution profile of products of incubation of McC^{519}, processed aspartamide-adenylate with aminopropyl decoration, without the enzyme and with MccH^{Hmi}, HinT^{Hmi}, and HinT^{Eco}.
