# Supplementary material for: Histidine-Triad Hydrolases Provide Resistance to Peptide-Nucleotide Antibiotics
Source: mBio. 2020 Apr 7;11(2):e00497-20. doi: 10.1128/mBio.00497-20 (PMC7157772; doi:10.1128/mBio.00497-20)
Supplement: FIG S4 [file mBio.00497-20-sf004.pdf]

SUPPLEMENTARY MATERIALS

Histidine-Triad Hydrolases Provide Resistance to Peptide-Nucleotide Antibiotics

Eldar Yagmurov[1], Darya Tsibulskaya[1,2], Alexey Livenskyi[2,3] Marina Serebryakova[2,4], Yury I. Wolf[5], Sergei Borukhov[6], Konstantin Severinov[1,7,8 *], and Svetlana Dubiley[1,2 *]

```
                               ** *                   * ‡*      ‡   *
HmiDSM14724a    ---------MSNCLFCKIRDGLIPAR-VVHRDDVCMAFEDINPQAPTHVVFIPLKHIP-TVNDIAQEDRE 59
TteBAA798       --------MAEDCIFCKIANKEISAD-EVWRDQDVVAFRDINPMAPVHVLVIPVSHIA-SLDQL--QDQS 58
EcoNCTC9094     VYPRRKKTMAEETIFSKIIRREIPAD-IVYQDELVTAFRDISPQAPTHILIVPNVLVP-TVNDVTAEHEQ 68
SteATCC33386    ----------MSTIFKKIIDKEIPAN-IVYEDNDFVAFRDINPQAKVHILVIPKKEIV-NLNEASDEDAL 58
HmiDSM14724     ---MSATDTQSSCFFCRLIAGREQAW-KIWEDEHHLAFLTPFPNAAGFTVVAPKKHLGSYVFSLEDA--- 63
PflA506         ----------MDCIFCSIARRTAPSH-LLWEDQTHMAFLSIFPNTPGFSVVIPKTHHGSYAFAEPDA--- 56
NkuDSM44524     ----MSSDHAPACVFCRIAAHDAPAH-RIWEDANHLAFLSIFPNTEGFSVVIPKAHRTSYVVEMEPS--- 62
SenNewport      ----------MTCIFCQIVEGKAPCH-KVWEDEHHLAFLSIFPNTDGFTVVIPKKHYPSYAFDLPPQ--- 56
ParGWA24037     MAEYEAKTKDDKCPFCEIAAGRFQTPGLFWQDDEFIAFLSIDLNTEGFSCVIPKEHYGSDVLKMPDD--- 67
MaePCC9809      ---------MKPCLFCEIASGLTSSH-ILHEDDKHLAFLDIYPATVGQTVVITKQHRSSYIFHLPEE--- 57
ParDG742        ---------MKDCIFCKIVKGEISSH-KIWENKEFLAILDINPNTKGMTLVLTKKHYSSYIFKMPSD--- 57

                              ‡        * * * ‡
HmiDSM14724a    AVGHLYTAAAQVAKARG--H-SEKGYRLVMNCNDDAGQTVFHIHLHLIAGRPMGWPPG----------- 114
TteBAA798       LAGKLILTASELARKLD--I-DDSGYRVVINTGPEAGQSVNHLHLHLLGGRSMSWPPG----------- 113
EcoNCTC9094     ALGRLFTVAAKIAQQEG--I-AEDGYRLIMNCNRHGGQEVYHIHMHLVGGRALGPMLAHKGL-------- 127
SteATCC33386    MLGKLQILIAKIAKDEG--I-ADDGYRVILNVNKDGGQEVFHIHYHILGGEKIGVLNSNK---------- 115
HmiDSM14724     EYTGLLLAARKVGLILDRAF-GTRRTGLFAE-----GMGIDHAHVKLTPLHGL-PE--GP--WQAVRSTV 122
PflA506         VLCDLVIAAKKTAKLLDKALKGVARTGMILE-----GYGVDHLHAKLFPMHGTGED--SA--FKKISSTQ 117
NkuDSM44524     EYTALHLAAREVARRLDAAFDGVARTGIMYE-----GYGVDHAHAKLFPMHGTAGN--QGQNWREVASPV 125
SenNewport      ALADLMLATQKVAKKLDKAFPDVSRTGMFFE-----GFGVDHVHSKLSPMHGTGDL--TH--WKPIESRQ 117
ParGWA24037     VLQRFIIASKKVAQVLENYFEDAGRVGLIME-----GTGIDHAHIKLVPMHGTGHMKQGE--WKQYLSGQ 130
MaePCC9809      EYSALLNFGRQVANILDIGL-KCQRTCLVME-----GMEIEHAHLKLYPIHTVKKSIAEE---------- 109
ParDG742        IYVRFLKAAKRVTKLLDKKL-KVQRTAMVME-----GMGVNHAHIKLYPLHGIKKK------FKEIYAPK 115

HmiDSM14724a    ----------------------------------------------------- 114
TteBAA798       ----------------------------------------------------- 113
EcoNCTC9094     ----------------------------------------------------- 127
SteATCC33386    ----------------------------------------------------- 115
HmiDSM14724     RTFYTRYEGYIA----------SHDGP----RMPDAELDALAERIR-KAAAEI 160
PflA506         DKYFERYEGYVS----------SHDAA----RADDKALAGLAAHIR-SFAW-- 153
NkuDSM44524     EGYFDRYQGYLS----------SHDHT----RAADTWLAEVAARIK-RAT--- 160
SenNewport      NKFFEQYEGYLS----------SHDHE----RADDEKLAARSA-IRHYTLD-- 153
ParGWA24037     EH--------------------------------------------------- 132
MaePCC9809      TIDLNEYNGYLC----------TLHGQ----RMSDWELAQVAQKIL-RVK--- 146
ParDG742        KVYFEKYPGYLT----------TQIGQ----RKKEDELEKLAKKIR-G----- 148
```

Figure S4. Conservation of the amino acid sequence in the Protein Kinase C Interacting protein-related clade of HIT proteins. Sequence alignment of HinT clade of HIT proteins (HmiDSM14724a, WP_044187632.1 of *Hyalangium minutum* DSM 14724; TteBAA798, ACZ41971.1, of *Thermobaculum terrenum* ATCC BAA-798; EcoNCTC9094, WP_096759427.1

of *Escherichia coli* NCTC 9094; SteATCC33386, ACZ09064.1 of *Sebaldella termitidis* ATCC 33386) and MccH clade of HIT proteins (HmiDSM14724, WP_044187428.1 of *Hyalangium minutum* DSM 14724; PflA506, AFJ55311.1 of *Pseudomonas fluorescens* A506; NkuDSM44524, WP_017574753.1 of *Nocardiopsis kunsanensis* DSM 44524; SenNewport, ECU0367860.1 of *Salmonella enterica* subsp. *enterica* serovar Newport; ParGWA24037, KKR61370.1 of *Parcubacteria bacterium* GW2011_GWA2_40_37; MaePCC9809, CCI22782.1 of *Microcystis aeruginosa* PCC 9809; ParDG742, KPJ57467.1 of *Parcubacteria bacterium* DG_74_2). Residues conserved in either of the two groups are shown in bold and underlined. Histidine-triad active site region is indicated by a red-shaded box. Conserved and partially conserved hydrophobic and polar residues forming the nucleotide-binding pocket of HIT proteins are indicated by an asterisk (*). Substitutions of the active site residues in MccH clade proteins are indicated by (‡). Red boxes mark residues of "inactive" MaePCC9809 and ParDG742 MccH-like proteins, that differ considerably from the MccH consensus.
