# Supplementary material for: Histidine-Triad Hydrolases Provide Resistance to Peptide-Nucleotide Antibiotics
Source: mBio. 2020 Apr 7;11(2):e00497-20. doi: 10.1128/mBio.00497-20 (PMC7157772; doi:10.1128/mBio.00497-20)
Supplement: FIG S5 [file mBio.00497-20-sf005.pdf]

SUPPLEMENTARY MATERIALS

Histidine-Triad Hydrolases Provide Resistance to Peptide-Nucleotide Antibiotics

Eldar Yagmurov[1], Darya Tsibulskaya[1,2], Alexey Livenskyi[2,3] Marina Serebryakova[2,4], Yury I. Wolf[5], Sergei Borukhov[6], Konstantin Severinov[1,7,8 *], and Svetlana Dubiley[1,2 *]

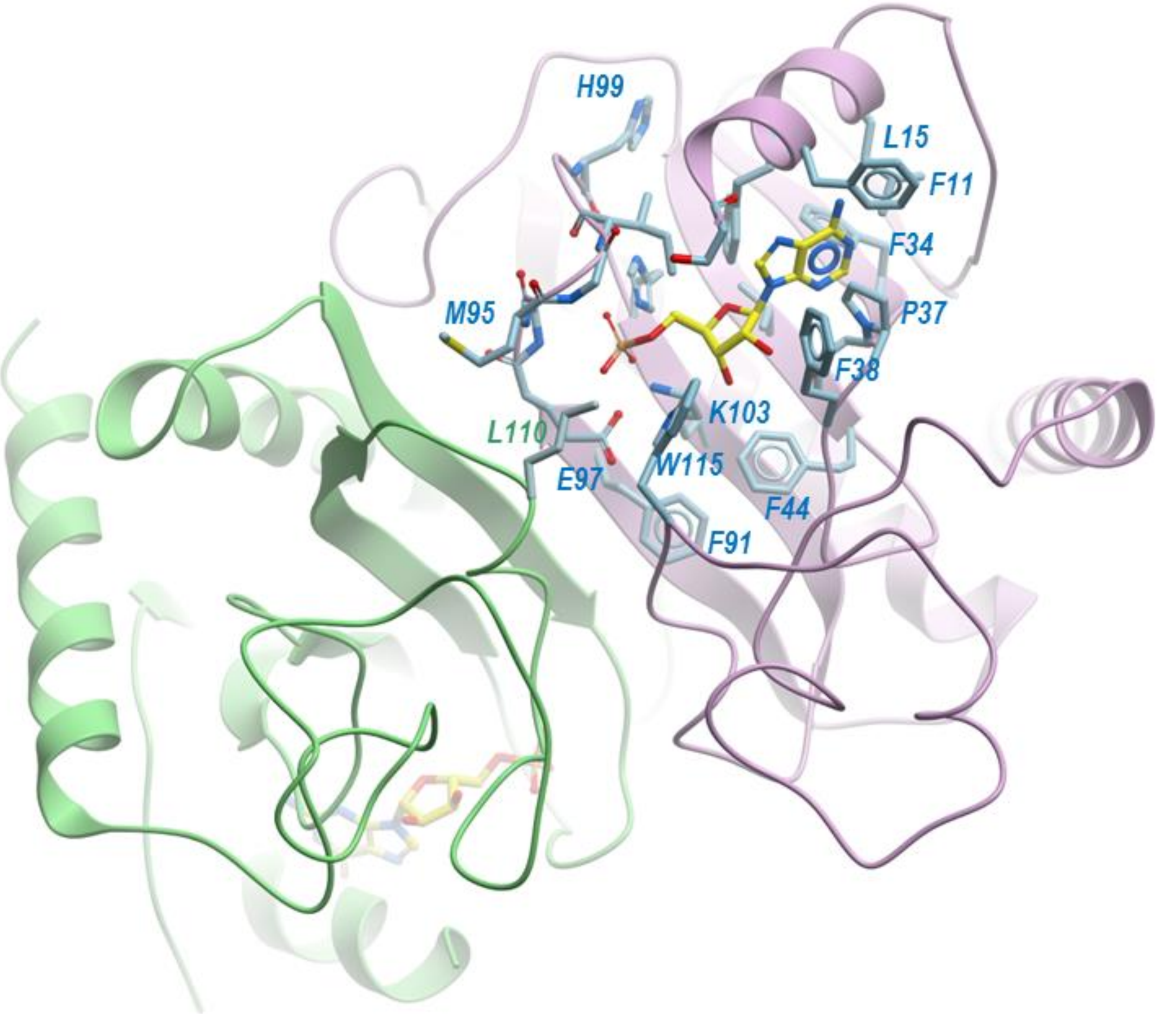

Figure S5. 3D-structural model of MccH^{Hmi} dimer in complex with AMP. The two monomers of MccH are depicted as light green and purple colored ribbons diagrams. Residues of the active site that form the substrate-binding pocket are labeled and shown in a stick representation.
