# Supplementary material for: Histidine-Triad Hydrolases Provide Resistance to Peptide-Nucleotide Antibiotics
Source: mBio. 2020 Apr 7;11(2):e00497-20. doi: 10.1128/mBio.00497-20 (PMC7157772; doi:10.1128/mBio.00497-20)
Supplement: TABLE S1 [file mBio.00497-20-st001.pdf]

SUPPLEMENTARY MATERIALS

Histidine-Triad Hydrolases Provide Resistance to Peptide-Nucleotide Antibiotics

Eldar Yagmurov[1], Darya Tsibulskaya[1,2], Alexey Livenskyi[2,3] Marina Serebryakova[2,4], Yury I. Wolf[5], Sergei Borukhov[6], Konstantin Severinov[1,7,8 *], and Svetlana Dubiley[1,2 *]

Table S1. Primers used in the study.

**Primer**	**Sequence 5’-3’**	**Procedure**
HmiA1_up	CATGAACGATAAAGCCACCATCGAAATCAAGAAAGATGAAAAGAAAGCGGAGCCGAAGAAAGTTGTGGTGGTTAAAACCAGCATCAAGGCCGGTCCGGCGGCGTTCAACTA	Cloning into pRSFDuet-1
HmiA1_down	AGCTTAGTTGAACGCCGCCGGACCGGCCTTGATGCTGGTTTTAACCACCACAACTTTCTTCGGCTCCGCTTTCTTTTCATCTTTCTTGATTTCGATGGTGGCTTTATCGTT	
HmiA2_up	CATGAACGAGAAAACCGCCCAGGAGAGCCAGAAGACCGAATCGCCGAAAGCCGAAACCCCGGCCAAAAAGGCCGTGATTGTGAAAACCCGCATTAAAGCGGGTCCGGGCGGTGGTGGCCTTGTGCATCCGATCGCCAACTA	
HmiA2_down	AGCTTAGTTGGCGATCGGATGCACAAGGCCACCACCGCCCGGACCCGCTTTAATGCGGGTTTTCACAATCACGGCCTTTTTGGCCGGGGTTTCGGCTT	

	TCGGCGATTCGGTCTTCTGGCTCTCCTGGGCGG TTTTCTCGTT	
HmiB1_F_NdeI	ATTTATCATATGAAAGACCACCTCTGGC	
HmiB1_R_KpnI	TTATATGGTACCTTATGCCTTGGGGACCTC	
HmiB2_F_NdeI	TATTATCATATGAACGACTATCTCTGGC	
HmiB2_R_KpnI	TTATATGGTACCTTAGGGCTGTGGCGTGGAGGG	
HmiP1P2P3_F_EcoRI	TTATATGAATTCCATGGCCATCCCCTTCTCCC	Cloning into pACYCduet-1
HmiP1P2P3_R_KpnI	TATATTGGTACCTTACGGGTGGGCGATGACG	
HmiB1_F_BamHI	ATAATTGGATCCGATGAAAGACCACCTCTGGCT GCTGC	Cloning into pET22_MBP
HmiB1_R_SalI	ATAATTGTCGACTTATGCCTTGGGGACCTCTTG ACGG	
HmiB2_F_BamHI	ATAATTGGATCCGAACGACTATCTCTGGCTGCT CCCC	
HmiB2_R_SalI	ATAATTGTCGACCACTCAGCCGGACCCTGGACT CAGG	
pBAD30_SalRBS_R_SalI	TATATTGTCGACT**TCCTCCTGTTA**GCCCAAAA AAACGGGTATGGAGAAACAG	Construction of pBAD30_SalRBS vector

pBAD30_F_HindIII	TATTATAAGCTTGGCTGTTTTGGCGG	
MccH_F_SalI	TTATAGTCGACATGAGCGCCACCGACACC	Cloning into pBAD30_SalRBS
MccH_R_HindIII	TTATAAAGCTTTTAAATCTCTGCGGCGGC	
HmiHinT_F_SalI	ATATAAGTCGACATGTCCAACTGCCTCTTCTG	
HmiHinT_RHindIII	TTATATAAGCTTTTACCCCGGCGGCCACCCCATG	
EcoHinT_F_SalI	ATTATAGTCGACATGGCAGAAGAAACTATATTCAGCAAAAT	
EcoHinT_R_HindIII	ATTATAAAGCTTTTACAGACCTTTATGCGCCAGCATT	
MccH_F_NdeI	TTATACATATGAGCGCCACCGACACC	Cloning into pET22(b)
MccH_R_XhoI	ATTATACTCGAGAATCTCTGCGGCGGCCTTG	
HmiHinT_F_NdeI	ATATAACATATGTCCAACTGCCTCTTCTG	
HmiHinT_RXhoI	ATATATCTCGAGCCCCGGCGGCCACCCCATG	
EcoHinT_F_NdeI	ATTATACATATGGCAGAAGAAACTATATTCAGCAAAAT	

EcoHinT_R_XhoI	ATTATA<u>CTCGAG</u>CAGACCTTTATGCGCCAGCATTGGT	
MccH_F44H_F	CCCAACGCGGCCGGC**<u>CAC</u>**ACCGTGGTGGCGCCCAAG	Mutagenesis
MccH_F44H_R	GCCACCACGGT**<u>GTG</u>**GCCGGCCGCGTTGGGGAACGGCG	
MccH_H99N_F	GAGGGCATGGGCATTGAT**<u>AAC</u>**GCCCACGTGAAGCTCACGCCGCTGC	
MccH_H99N_R	GAGCTTCACGTGGGC**<u>GTT</u>**ATCAATGCCCATGCCCTCGGCGAACAG	
MccH_H101N_F	CATGGGCATTGATCACGCC**<u>AAC</u>**GTGAAGCTCACGCCGCTGCACGG	
MccH_H101N_R	GCGGCGTGAGCTTCAC**<u>GTT</u>**GGCGTGATCAATGCCCATGCCCTCGG	
MccH_K103H_F	ATTGATCACGCC**<u>CAC</u>**GTGCACCTCACGCCGCTGCACGGCCTCCCC	
MccH_K103H_R	GCAGCGGCGTGAG**<u>GTG</u>**CACGTGGGCGTGATCAATGCCCATGCC	

Sequences of restriction endonucleases recognition sites are shown in plain typeface and are underlined; Sequences corresponding to ribosomal binding sites are highlighted in bold; Codons corresponding to mutated amino acids are underlined and highlighted in bold.
