# Supplementary material for: Histidine-Triad Hydrolases Provide Resistance to Peptide-Nucleotide Antibiotics
Source: mBio. 2020 Apr 7;11(2):e00497-20. doi: 10.1128/mBio.00497-20 (PMC7157772; doi:10.1128/mBio.00497-20)
Supplement: FIG S6 [file mBio.00497-20-sf006.pdf]

SUPPLEMENTARY MATERIALS

Histidine-Triad Hydrolases Provide Resistance to Peptide-Nucleotide Antibiotics

Eldar Yagmurov[1], Darya Tsibulskaya[1,2], Alexey Livenskyi[2,3] Marina Serebryakova[2,4], Yury I. Wolf[5], Sergei Borukhov[6], Konstantin Severinov[1,7,8 *],

and Svetlana Dubiley[1,2 *]

```
>CON.1
----xxxxDCLFCKIVAGEIPATVVRETExTLAFRDINPQAPTHVLVIPKxHYxDAAxLAAALxADVLxxAxxVAxxEG--IxxxGYRLVFNTGxxAGQTVFHVHAHVLGGRxLxWPP-------G----------------------
>CON.2
-----xxExxIFxRIIxGEIPAExVxExDxVIAFxDIxPQAPVHVLVVPKxxYxNVxELAAGLLAEMVxVAxxIAxExx--x--GEFRLVFNTGxxAGQTVFHVHAHVLxGxLxExxx-------x----------------------
>CON.3
----xxxxDCLFCRIVAGEIPAxIVAETDxVVAFRDIxPQAPVHVLVVPRxHxxDVAQLAAALLAEMVxVAQQVAxxEC----xGQFRLIFNTGPxAGQSVFHVHGHVIGGxxLGWxP-------A----------------------
>CON.4
----xxxxDCLFCKIVAGDVPADVVAETEHTVAFRDIDPQAPTHVLVIPRxHEPDVGSLAAAAAVxLLxxARRVADxxG--xx--SYRLVFNTGADAHQTVFHCHGHVLAGRxLGWPP-------G----------------------
>CON.5
----xTxxDCLFCRFVAGEIPPDVVAESExSLAFRDINPQAPTHVLVVPRRHVPNAGxLAAADLADVxxLAxxVAExEG--L-xxxYRLVFNTGAxAGQTVFHAHLHVLGGRxFxWPP-------G----------------------
>CON.6
----xxxDDCLFCKIVAGDIPADVVHxxxTTVAFRDLNPQAPTHVLVVPRSHYxNAAALAxxxLADLxxxAxEVAExEG--Lx--GYRxVFNTGAxAGQTVFHAHLHVLGGRxLxWPP-------G----------------------
>CON.7
----xxxxDCLFCKIVAGEIPxDIVHxxERTVAFRDINPQAPLHVLVVPRDHxPNAAAxAxAxxAELVTTAAAVAxAEG--YD--DYRLVFNTGAGAGQTVFHTHLHLLAGRxMTWPP-------G----------------------
>CON.8
-------xDCLFCKIVxGEIPAxIVxRxDxVxAFNDIxPQAPTHILIIPxxHxENAAEVAxxLxGELFxAAGxIAxExG--LD--GYRxxFNTGAxAGQSVFHAHLHLLGGRxFAWPP-------G----------------------
>CON.9
-----xxxNCLFCKMxxGxIxVTxVxENDxxFVIxDIxPKAPVHxLVIPKKHYxNxxxxxVxxIGxLxxxxxxLxxxEN--Lx-xGFRLVxNTGADAGQSVxHLHLHLLxGRSxxWPP-------G----------------------
>CON.10
------xxxCIFCKIVxGEIPADVVYEDExVIAFRDINPQAPVHVLVIPKxHIxSxxEVx-xxxxxLMxxAxxVAxxLG--LE-xGYRLVINxGxxAGQTVxHLHVHILGGRxFxWPP-------G----------------------
>CON.11
------xxxTIFxKIIxREIPADIVYEDDxxLAFRDINPQAPVHILIIPKKPIPxLxDAxxELLGHLxLxAxKIAxQxG--Lx-DGYRVVINxGxxGGQTVFHLHLHLLGGRxMxWPP-------G----------------------
>CON.12
-----MxxDTIFxRILRGEIPCDEVYxDExCLAFRDIxPQAPVHILVIPRKPIxSLxEAxxELLGHLLLVAAKVAKxEG--LE-x-WRTVINTGAEAGQTVFHLHVHVIGGRPLxWPP-------G----------------------
>CON.13
--------xCIFCKIAxKEIPxKVVLEDEHxLAFHDLNPQAPTHVLVIPKRHIAGLAEAxPEVLGRxLLGAxQVAxKLG--Ix-EGFRTVINSGANAGQTVFHLHVHVLGGRxMGWPP-------G----------------------
>CON.14
-----xxxDCLFCKIVAGEIPAxxVYEDExxIAFxDINPQAPxHVLIIPRxHIxSLAxAxxxLLGHLMxxAAEIARxQG--LxxxGYRVVVNTGxDGGQTVxHLHLHLLGGRxMxWPP-------G----------------------
>CON.15
------MDxCIFCKIIAGExPxxILYQDELVTAFxDxxPIAPVHILIVPNxHIESVNxVExxLLGHMIxVARxLAxxxG--LxESGYRLVINTGPNAGQSVFHLHMHLIGGRxMPFxx-------x----------------------
>CON.16
------MxDCIFCKIVxGEIPSxxVYEDDxVLAFxDINPxAPVHILVIPKKHIxSLxxIxxELIGHIxxxIxKIAKExG--IxExGYRVVxNCGxDGGQTVxHLHFHLLGGRxLxWPP-------G----------------------
>CON.17
------MxDCLFCKIxxGEIPAxKVYEDDxVLAFxDIxPQAPxHxLIIPKKHIxxxxDLxxELIGxIxRVAxxIAREXG--IxxxGFRIVxNNGAxAGQSVFHIHFHLLGGRxLxWPP-------G----------------------
>CON.18
------xxDCLFCRIVAGEIPSxxVYEDDxVYAFxDVAPQAPVHILIIPRQHIPSxxDLxEAVVSHLIVVAxDIAQxEG--IAESGYRLVxNVGxDGxQSVDHLHFHLLGGRxLEWPP-------G----------------------
```

```
>CON.19 (Hit Hmi, 202 sequences)
------xxDCLFCKIVxGEIPAxVVYEDDxVIAFxDINPQAPxHILVIPKKHIxTLNDLxxxLLGxMxxxAxxIAxxxG--xxExGYRxVxNCNxxGGQTVYHIHLHILGGRxMxWPP-------G---------------------
>CON.20
-------MxCIFCxIAxGEIPADxVYKDEDVVAFRDINPQAPVHVLIIPxRHIASLxDLxDxLAGxMxxVANELARxxG--IxESGYRVVVNSGxEGGQVVxHLHLHLLGGRQLSxxL-------G---------------------
>CON.21 (HinT Eco, 213 sequences)
-----MxEETIFSKIIRREIPADIVYQDDLVTAFRDIxPQAPxHILIVPNxLIPTVNDVxxExLGRMFTVAxKIAKQEG--IAEDGYRLIMNCNxHGGQEVYHIHMHLLGGRPLGPML-------x---------------------
>CON.22
------xxxTIFxKIIxREIPAxIVYEDDDIIAFxDIxPxAPVHILIIPKKEIxTINDIxxELIGKMxLxxxKLAKELG--IDxxGYRVIxNxNEDGGQTVFHIHFHLLGGEKLGxxx-------x---------------------
>CON.23
-----xxxxTIFxRIIxREIPADIVYEDDxFIAIRDIxPKAPVHLLVIPKKxxxRLxDLxD-xMGxLxLxAxRVARxxx--Lx--GYRLxVNVGExGGQEVFHVHIHILGGxxxx--------------------------------
>CON.24
--------xCVFCKIVNKEIPAKIVYEDExxMAFHDINPQAPVHILIIPKEHIPxxxxLEEKIIGHIxLxANEIAKxLG--IxETGYRIIVNxGxDxGQEVFHIHxHLLGGxxLGxxI-------x---------------------
>CON.25
------MExCIFCKIINKEIPAKIxYEDDxVIAFHDIxPxAPVHILIIPKKxIPTLxDVQEELIGHIHxVAQxLARxLx--L--xGFRLVxNCGKEGGQEVFHIHYHLLGGxxx---------------------------------
>CON.26
------xxDCIFCKIVxKEIPxEIVYEDDxIVAFKDIxPxAPVHILIVPKKHIxSVxxLxxxLIGKMIxxAKKIAxExG--IxxxGYKLIFNVGRxGGQxIxHLHLHLLGGxxxxxxx-----------------------------
>CON.27
-----QxKDCIFCKIIRKEIPSKGVYEDDLVYAFHDINPVAPVHILIIPKKHIFGIQxLEPELVGHMFYVARKIAEELG--LANGGYRLVFNVGKDAGQSVFHLHLHLIGGRSMSWPP-------G---------------------
>CON.28
------MxxCVFCEIIAKKMPADFxYEDDEIVAFxDINPQAPVHILVVPRKHYPTLxDxKxELLGKMILVANEIAKKFN--IHxxGFRLVFNxNREAGQSIYHVHLHLLGGRIMMWPP-------G---------------------
>CON.29
----x-xPDCLFCRIVxGEIPAxIVYRNDHVLAFRDIxPVAPxHVLIIPxKHIASLNDLxxEIAGQILLAAxxVAExLG--IxESGYRLVFNTGxDALQSVFHIHGHLIGGxxMGWPP--x----G---------------------
>CON.30
------MxDCIFCKIVAGELPSKxxYEDDxIVAFxDINPxAPVHVLIVPKKHxxNLADAxxELLGKLxLVAxEIAxKxG--Ix-xGFRIxTNxGxxAGQxVxHLHYHLxGGxxxxx-------------------------------
>CON.31
-----xxxDCIFCRIVAGEIPAxxVYRDDxVVAFxDIxPQAPVHLLVIPxQHIxSLxxAxxxxLGxLxLVAAEVARxxG--LExxGYRVxxNxGxxAGQxVFHLHFHILGGxxxxxxx-------x---------------------
>CON.32
-----xxxNCIFCKIVxGQIPSKKVYEDDEILAFHDINPxAPVHFLIIPKxHIxSLxxxxxxLLGRMMxLAPRLAxExG--xx-xGFRTVINTGxxGGQEVYHLHIHVLGGPRPWxxx-------x---------------------
>CON.33
------MSDCIFCKIITGEIPxKKVYEDDNILAFHDINPAADVHVLVIPKKHIASLNExxxELMGKFMLxIPKIAKxLG--Lx-xGFKTVFNTGKEGGQMVFHxHAHILGGxxxxxxx-----------------------------
>CON.34
------MxDCIFCKIVxGEIPAxxVYEDDxIIVFKDIxPKAPVHLLVIPKxHIxxLxELxxxLMxHMMxxLPxIAKxQG--Lx-xGFRTIINTGxGGGQEVxHLHIHILGGxxxxxxx-------x---------------------
>CON.35
------MNDCLFCKIVxGxIPxNKVYEDDxVxAFRDIHPKAPTHVLVIPKQHIxTLxDV--ELLGxLMxRVQHIANxIx--xxExGYRVVINxREGGGQEVFHLHxHILGGKxLPx-x-------x---------------------
>CON.36
------MSDCIFCKIxxGEIPCxKIYEDDxVLAFxDIHPxAPVHVLIIPKRHIxTIxDIxxxLxGxMIxAAxEVAKxxx--IxExGYRLVxNCNxDGGQVVFHIHMHLLGGRKLxxxx-------x---------------------
>CON.37
-----xDxxCLFCKIxAGEEPSEKVWExxEFLCIKNKYPVAPVHVLVMPKxHIEKxEVxTGxFWxKMMxAVFEVVxxLG--LD-KGYKLVNNGAGY--NHFEHEHxHILGGSKxEPGG-------x---------------------
>CON.38
------MIxDVFCKIIKGELKSExVYKDxDFxVIKDINPQAPVHLLIIPxKHFExIxxFKxxLLGKxLLIADKVAxxxG--Lx-xGYRLIINEGxDGGKLVPHFHIHLLGGKRLGxKI-----------------------------
>CON.39
------MxxCIFCKIxxGEIPSxKVYEDEDMIIIKDINPQAKIHYLLIPKEHFAxIxEMTEExLGRxLKKLSxxxDELG--LQ-NGFRLVSNKGEDGxQSVxHLHIHILGGEKLSxxM-------x---------------------
>CON.40
------xxDCIFCKIVEGxIPSKKVYENDxVLAFHDIQPAAPVHVLIIPKKHIASMNDVxxELIGEIHxAAQQIAxELG--IAESGYRLINNCGxDxGQxVFHLHYHLLGGxKLxxxx-------x---------------------
>CON.41
-----xxDDCIFCKIAxGEIPSxxVYEDDxVxAFDDIxPxAPVHVLIVPKxHYxNIxDGVP-xLxxLxxAVxEVAxxKG--Ix-EGFRVIxNTGxDAGQTVxHLHIHVLGGxxMxxxx-------xx--------------------
>CON.42
------MEDCIFCKIVRRELPSDIVYENExVLAFKDINPMAPTHVLIIPKxHLxSLNDLxExxLxxIxxAIKEVAKIxG--VYExGYRVISNCGEDGGQVVxHLHFHILGGKKLxxxI-------x---------------------
>CON.43
-----xxxDFYCDEVLSGKTxVxxVxETDxVLAFHHTRPFWPVHIVVIPKxHIxSLIxLxxxLLxELLxVVRxVAAxVx--xEHGACRVxTNLGxY--QDSKHLHWHVxxGxxxxxx------------------------------
```

>CON.44
----xxxGxxAFEx-LSxLLPVKRVxENDxVIAFWHPxPxWExHILIVPKKKIRxFxSLNxExIxEIFKxAKxVVxQxG--W--xEYTILVNGGDx--QDVPQIHFHLCSGxExxGxx------------------------------
>CON.45
--------MxIFxKIVxGEIPxNKVLENDxFLAFHDINPKAPVHILVIPKxHxxxFQxVxPELMxxMTxFIQEVAxxLG--IDESGYRLITNxGxDGGQEVxHLHFHILGGxKLxWxx-------xx---------------------
>CON.46
------MDDCIFCKIVKGExPxxIEKETDNLIVIxDINPxAxIHLLIIPKKHIRDIxx---xLWxEIKxVALxLAKEKx--L--xGFRLVxNxGDA--AxVxHMHIHFLGxITxDRxI------------------------------
>CON.47
-----xxxxCIFCKIIxxxIPSxIIxENDxVIVIKDIxPKAPIHYLIIPKKHIxDIxxLxxxLxxxMLxMAxxLSxxLx--xx-xxFRLVINNGxxAGQxVFHLHxHFLAGKxxxxxx-------xx---------------------
>CON.48
-----xxxDCIFCKIIxGEIPSEFVYExExVVVFxDINPxAxVHLLVVPKKHIxSIxEIxDxxLxxMxxxVKEIVKExx--Lx-xxYRLVxNGGxx--QxVxHLHxHLLGGxxxxxx-------------------------------
>CON.49
-------xxTVFxKIIxGxLxxEKVFENENILAIKDxxPxAPVHLLIIPKKEIxxLQSIxxELIxEIxxIAQxLAEEFG--Ix-DGYRLIxNNGxxAGQSVFHLHFHLIGGRxLGxIA------------------------------
>CON.50
-------xDCVFCKIIxGEIPSExVxEDExFIAFRDINPVAPxHILVIxKKHVxxIxELTxxxMxKLFELIxEIxExxG--IExxGYRIVxNxGKDAGQEVxHLHFHIIGGRKLGxIG------------------------------
>CON.51
----xYDDxNIFAKILRGEIPxxxVYEDEHxLAFHDIxPQAPVHVLVIPKGxYVSxDDFxxAEIxGFxRAVGxVARxxG--LxxxGYRLIxNxGxxGxQEVPHFHVHILGGRxLGxML-------x----------------------
>CON.52
----MYDxNNVFAKILRxEIPCHKVxENEHALAFxDINPQAPIHILVIPKxAYxDFHDFxSAEIxxFWKLVNDIIxxxS--ISxxGFRIISNSGxDGNQDVPHFHVHLLGGKNLGRMI-------N----------------------
>CON.53
-----YNNNNVFAKIINKNLPAExIYEDEQVLAFxDIAPxAPVHIIVIPKNEYIDYxDFIxAEIxxFFxKIxDIANxxG--LDKxGYRLITNxGEDSxQxIFHFHFHIIGGEKLxGLI------------------------------
>CON.54
------MSDCLFCKILKGEIPSTRVYEDESVxGFRDIxPMAKEHYLFIHRNHTKNVNEMSxxELSDVFxAISKYTESNE--Lx-NGFRVVTNIGPDGGQTVFHTHFHVLGG-EKLKGF-------G----------------------
>CON.55
-------xxCIFCDIIxGKxxGHIIYEDExHVAFLDKYPIDxGHTLVIPKKHHExITDMxxxEVGxLFSxVPKIAKAILxATGADAFSIAQNNGRAAKQIIPHVHVHIIPRYNxKGxx----WTRx-IxxDDELxELAxKIRxxLx--
>CON.56
------xxDCVFCxIVxxKxPAHIVYEDxxxIAFLDKYPxTxGHLxIVPKxHYRxIFDLPxxxIGExFxLAKxIAxAxxxVLGAxGVxLxMxxxxxxGQEIxHAHLxVIPxxxx--------xxRx-xLxxEEGxxVAQLLREALxx-
>CON.57
-------MDCVFCRIVRGExPAYVVYEDDxVVVILDKYPVSxGHLLVMPKRHYxxIxDxPxDxLxRxFxVAxxIAxxxxxxLGAPGVNIVTNxGxEAGQVVFHFHIHVIPRWGxxxx-----xxRH-ELTEEEAREVVExLxxxIxE-
>CON.58
--------MCLFCxIIxGxEEGYIVYxDExVxAFLDKFPISPGHTLVVPNxHFENFLxTxEDxLxxLxxxxKxIAxAVKxALKADGIRILTNVGRSAGQVIFHxHIHIIPTWDxxxx-----FxxF-xPRKxQxKEYYExLQxVIxxx
>CON.59
------xxxDIFxKIIxREIPAxIIYEDDKVIAFxDxxPxxxGHFLVVPKxxSxNLxExDDExxxYLIxxARELAxExVxxxGxxGFKLxINNGxxAxQxVFHTHIHIIPYxx-----------------------------------
>CON.60
-------xDCLFCKIINGEIPSxTIYEDDxVxVFLDINPxSxGHTLIIPKKHYxDLxDIDxExLxHIxxVAKxIxxLLxEKLNxDGLTLxQNNGx--xQEVKHYHLHLIPxYxxxxxx--------------xVEEVYNxLKx-----
>CON.61
------xxDCIFCKIINxEIPSxxIxENExVFAFLDIxPxSDGHxLVIPKKHxxNFSxTDDxxLxEVxxxxKxVxxxLxEKLxPxGFNYISNQGxxAxQxVFHYHxHIIPKYxKxEG---Fxxxxx-xxxxxxLxxVxxxLxx-----
>CON.62
------MxDCIFCKIVxGEIPxxxIYEDExTLAFLDxxxDVDGHxLVIPKKHxxNILDCDDxxLxxIMxxVxxVSxHLVxxxGYxGVNILNANGxxAxQSVxHLHFHIIPRKxxDGIx---xWPxx--GxxxxLxxxxxxLxxx----
>CON.63
------MxxCIFCKIVxGExPSxxVYEDExVxAFLDxxPxTxGHTLVIPKxHYxNIFDIPEExLxxVIxVxKKIAxxxKExLGxxGIxIIQNNGxxAxQxIFHFHIHIIPRxxxDxxx--xxxxxx-xxxxxxFxxxxxKIxxx----
>CON.64
x----IMxDCIFCKIVKxEIPCYKVYEDDLVLAFLDINPLNVGHTLVIPKQHSEDLLxMDDExNxRVLKVCKKIAxSLKK-LNxxGINIYSAIGSDAGQVVFHTHFHVIPRFKGDxFK---RxxxI-ELSxDEFxDLSKKISQNI---
>CON.65
------MxDCIFCKIIxGEIPSxKVYEDDxVxAFLDISQVTxGHTLVIPKxHxxNIFExDxExAxxIFxxVPKIARAIKxxxxxxGMNILNNNGExAGQSVFHxHIHLIPRYxxDGFx--xxWxxH-xYxxExLxxIAxxIxxxLx--
>CON.66
------x-xCIFCKIxxxEIPxxxIYEDDxxLAFLDISQxTxGHTLVIPKKHYDNFLExDxETLxHxxxVAQxLAxxIxxxLxAxGINILTNxNExAGQTVxHFHIHIIPRYxEDxxx--IxFx-x-x---xDLDxIxxxIxx-----
>CON.67
-------MxTIFTKIINREIPAYIIYEDDLVIAFLDISQATKGHTLVVxKxxYxDIFEVPExVxxHLFxVVxKISxxLxKxFxxxGINLLNNNGxVAGQTVFHYHVHIIPRYxxDEIx--FxxxNN-xLxxxDYxxxxxxIxx-xLx-
>CON.68
------MxExIFxKIIDGEIPSYxVYEDDxVxAFLDxxPVSKGHTLVIPKKxxxNIYExDxETxxHIxxALxKVANAVKxAFNPDGLNIxQNNGEYAGQSVFHLHFHIIPRYxxDGFx--YKWExx-xLxxExxxEIAExIxxxLx--

```
>CON.69
-------MDCIFCKIIAGEIPSxKVYEDDDFIAILDINPVxxGHTLLIPKKHFxNIFDTPDDVGxKIYPVLxKLAxAIKEALxCDGINIVQNNExAAGQEVFHSHIHIIPRYENDxIK--FxxxxK-xYxSxEMxKxAEKIKxxIx--
>CON.70
x----xxxECLGCRLANKxExVHVVYEDDYVxCFLDHxPFNEGHTLILPKxHxxEVDELDxxTAxAIMxASxLISKAIKxLYxPDGITICQNGGV--FNELTHYHMHVVPRYKxQxFY----xxxxxxxxxxxLxETxxxLxxxIxxL
>CON.71
-------MDCLGCRIANGIEPLNIVYENExITCVLDIAPFNEGHTLILPKKHYxDVEEMDxETAYAIMDASxKLSxVLKxLFxPDGISICQNGGx--FNDLTHYHMHLIPRYEGDGFx----WSEPPHGAExRLxETRxKIIxALxxx
>CON.72
x----xxxxCLGCxLAxKxI-TNTVYENDLVTCILDIAPLNEGHILILPKxHYxDVDDLDEITAxEIMKTSAVLAKLLKxQFQPDGITVIQNGGK--FNDLNHYHMHIFPRYESDGFA----WVEPTTNAKGRLxETREKLIxLIxQQ
>CON.73
------MENCxFCKIINKEKKANIVYENDLVCCFLxEEPINEGHMLIVPKKHxLDLDQLDxExAIEIMKISKIMVRVLKDTYKxDGYSIMQNGGS--FNNVGHYHMHLFPRYKGDGFS----WSYGEDx--xTLEVVSKKIxQQLKxx
>CON.74
------MxxCIFCEIISKKxDAYIIYENDYVCCFLDKYPINKGHILVVPKxHYxEFxEVDxESLxxVIxxAQKIAxALExxxxTDGITIMQNNGI--FKDVEHYHMHIIPRFxDDGFS----WVEPxxVxxEDFxSLxxxLRxxLxKx
>CON.75
--------MTIVEQIVxREIDAVIVYESxxVIAFADHDPINFGHILICPxxPYxTFIDLPExIxxEIxxVARDLYRRIExxFxPDGISFLQNNGx--FNELxHYHLHIFPRFxGDxFG----WxSSLGIQSx------ExLRESLxxL
>CON.76
-------MxCIFCxxIxxx---xILxQTEHFxVVWDIDPIQxGHLLIISKxHYxxIxELxxxxxxELIxLExxLIExLExxxxIxGVTIAxNNGMDxG---THFHVHLIPRYxxDGF-----WDxx-xVxxxxx--xLxxFxxxLxx-
>CON.77
M----xxDDCIFCKIANGEIPSxTIYEDDDFRVILDxxPASKGHALILPKxHYxNIYELxDExAAKVxxLAKKIAxxMKxxLxCDGxNIVQNNGEAAGQTVFHFHMHLIPRYxxDxxx--IxWxPx-xxxxEExxEIxxxIxxxx---
>CON.78
------xxxCVFCxIVAGxxxAxxVxEDxxxVAFLDxRPLFxGHVLIVPRxHVxTLxDLPxxxVxPFFxxVQRLAxAVExALxAxGSFVAxNNxV--SQSVPHLHVHVVPRxKGDGLR--GFWPRx-RYDxxExxxxAxRIRxALxx-
>CON.79
------xxxCxFCxIxxxxxDxxLVxRxxxVVPVLxQRxxNPGHxLVLPVxHVTxLHxVPxDxLxEIFxVxARVTxAVRDAFGAVGSxVVQNNxx-PGQxLxHxHVHVIPRxxGDGFx--xxDPxx-xxxxxxRxAxAAxLRRxLxx-
>CON.80
x----xxDSCILCxINQDxLxHFxIAExxxFKAVLDxYPITEGHILILSRSHxSHLEQLxDxEYxELFxFARxLGxRMxxVMDxVDYNLIVNNGx-xGQHIPHVHIHLIPRRKGDSLx--FYWTRF-PxxxxxRLxKVxxKxxxx---
>CON.81
-------MxCIFCxIxAGxLPAxxVYEDExxIVLMDIYPLxxGHVLIIPRxHxxxLxELxxxxRxHLxxLAxRVxxAxRxAxGxxGxNLLINDGxxANQHVPHVHLHIIPRRxGDxxx--xxxRFG-xxxxxxLxxxAxxLxxxLxx-
>CON.82
------xxxCVFCxIVAGxAPAxxVYEDDxxLAFLDIRPITRGHTLVIPKxHxxDLxDLxPxxGAxMFxxGQRIAxAxRxSLxADGxNLxINDGRAAFQTVFHxHLHVVPRxxGDKLx--FAKGxL-xRRxxDxExTAxxLRxALxx-
>CON.83
-----xxxxCVFCxIVxGxAPASxVYEDDxVLAFMDIxPVTPGHLLVVPRxHAxxLxDLDxxxGAxMFxVGQRIAxALRxSLxCEGVNLFLADGxAAxQEVFHVHLHVIPRxxGDGFx--Lxxxxx-xxxRxxLDxxAxxIRxALxx-
>CON.84
-----xxxxCIFCRIVAGEIPASxVYEDxxTIAFMDLxQVNPGHVLVxxKxHxxTIYDLDxExAAAVMRTAxRVARAVxxAFxPxGLTLxQANGxAGxQTVxHFHLHVxPRxxxDGIx--xxWPxK-xPxxxxLxxxAxRLRxxLxx-
>CON.85
-----xxxxCxFCxIVxGxAPAxIVREWxDALAIxPxGxVTxGHVLVIPRxHVxDxxxDPx-VTxxxMxRAAELAAExxxx-----xNLITSxGxxATQTVxHLHxHLVPRxxGDGLx--LP-----WTxxxxxxxxxx---------
>CON.86
-----MxAxCVFCxIVAGxAPADVVREWDDVIAIRPLxPVTPGHVLVIPHTHVxDVGQDPx-VSARTMACAAxLAGxLP-A-----ANVITSKGxAATQTVxHLHLHIVPRTAGDxLP--LP-----WTPQQxxx-----Ax------
>CON.87
x----xxxxCxFCxIVxxxxPARxVYRDxxVVAFFPxxPATxGHTLVVPRxHVxDIWxLxxxxAxxLxxAVLRVAxAIRxALxPDGLNIIQSNGxAATQTVxHLHVHLVPRWxGDxMG--xIWPxx-xxSxxxxDxxxxxIRxAxxx-
>CON.88
-------xxCxFCxLIxxxxxAxWVARxxxAxAFxPLxPLAPGHTLVVPxxHxxDIxDAPPxxLAATxxLVQRVAxAMRxALxAxGVNILSASGPGSEQSVPHLHFHVVPRWxDDGIS---TWPxx--SxHRxxGDPxxxLAxALx--
>CON.89
-------MTCPFCSIVxGDEDAxILDETDETLAFAPLxPxSEGHLLVVPKxHYESLFDIPExTLxxVxxHARxIAxRLRxx-GFDGVNLLHASGEAAQQSVPHFHIHLAPRRSDDxLD---LWPES--YEESxxxxxYExIRxxLE--
>CON.90
----YExYxCPFCxxVxGIEQxDIIYQDExVTAFIASxPNNKGHVLIIPNxHYENIYDLPxExAxxIHxxAKxVAIAMKEVYKCDGVSxRQHNEPxGNQDVWHYHLHVFPRYxxDNLY--xTx-xx-xSxPEERxxYAEKLRxxFxx-
>CON.91
----xxGYxCPFCxLAxGDxxxDLVYQDDxVLVFIAxDGPHxGHVMIxPxxHxExLYDLPDxVxxRIxxxTRxVALAMKxAWNPEGVSTRQHNEPAGNQHVWHYHxHVFPRWxDDxLY--xxx-RH-PVDxExRAxKAxELRAALxx-
>CON.92
----xxxYxCPFCxLxxGxxxxDVVxRTERAxAxIAPRPxNxGHVLVIPxxHxENLYDLPxxxGHAVxDLVQxVAVAMRxxYGCxGVSTRQHNEPAGxQDVWHxHVHVFPRYxGDxLY--xxx-xx-xVxxEERxPYAxxLRAxLxx-
>CON.93
----MPGYxCPFCGIAxTLPESAVVLVDxNVFALVPTHGGIKGNCLVIPRxHYENVLDIPDxLGxDFFRATRRLAxAMRxVFxCEGISTRQHNGPAGNQDVWHYHLHVFPRYxNDGLY--GGQ-KV-PYSTEERIELAARLRAALH--
```

```
>CON.94
x----YDxxNIFAKILRGEIPxxKVYEDDxTLAFMDIMPQAxGHxLVIPKxxARNLLDxxxExLxxxIxxVQKIAxAVKxAFxADGIxIxQFNxxAAGQTVFHLHFHIIPRxxGxxLx--xHx-xx-MxDxxxLxxxAEKIxAALxx-
>CON.95
x----YDxQNIFAxIIRGEAPCYKLYEDDDVLAFLDIFPQSxGHxLVIPKSxARNILDVDxEALAQVMAVVQKLxxAIVDELQPDGVQVAQFNGAPAGQTVFHIHVHIVPRWxGExxG--xHA-xx-xADPAELEALQxRLxxRLxx-
>CON.96
x----YDxxNPFARILRGELPAxxVYEDxxVLAFMxxxxxxPGHVLVIPKxxARNLLDIxxxDLxxVMxVAQRVGxAQxRALGxxGFxIxQNNG--xGQxVxHLHVHVIPxxxxxPx-----x-xx-xxxPxELEAMAxRIRAAMxx-
>CON.97
-------xxCIFCRIVxGELPCHKVYEDDxTLAFLDIxPASxGHTLVIxKxHxxDIFDxxPExIAAVAxxxQRVAxxLxxxLxPDGLNIxQxNGxAAGQTVFHYHMHLIPRWxGDxxx--xxWxPx-PxDPxELxxLAExLRxxLxx-
>CON.98
-----xxxDCLFCxIVAGDIPSxxVxEDDxTxAFMDINPxxDGHxLVIPRRHxxDLLExxxxDLAAVxxxAQRVARxxxxxLGADGVNLLNxCGAxAWQTVFHFHLHVIPRYxDDxLx--LPWxPx-xGDxDxIxxxxxxLxxxxx--
>CON.99
x----xxxxCVFCxIVxGRxPAxxVFEDEHTVAFLDIxPAxPGHTLVVPRxHAADIWEIxxxxxAxVMRxVHRVAALLxxxLxPDGLTIxQANxxAGWQDVFHLHVHLVPRxxGDxLx--xPWxxx-xAxxExLxxVxxRLxxxxxx-
>CON.100
------xxxCIFCxIVxGxIPSxxVYEDDHxxAFMDIxPAxxGHTLVIPKRHVRDIxELxxExxSHVMxAAxxVADLIRxALxPxGINLFHxNGAAAxQSVFHFHMHLIPRWxxDxLR--xPWExS-xGDxDxIxxIAxxIxxxx---
>CON.101
x----xxxDCIFCKIVxGEIPxFKIYEDERxLAFxDINPIxPGHTLVIPKxHAENIxEIxPEDLAAVxxTxQRVAxAIKxALxPxGIxILQLNGxAAGQVVxHYHIHLIPRxxxDxxx--xxWExx-PGDMxxIxxxAERIxAALx--
>CON.102
-----MxDDCxFCRIVAGDxxAHVLYEDDxTIAFLDxNPAVxGHTLVVPRxHxxEVLxxDxxxxAAVFETVRTVAxALExALDPDGFSVFHTSGPLVGT-VDHAHVHLVPRFxDDxVS---LSLxR-xLxxxxAxxLxxRIRxxL---
>CON.103
------MSDCVFCxIVxxExPAHRLYEDERSLAFLDxEPAxxGHVLVVPKAHHETLTDMPExLAGAVFxTVxRVAxAIESAxxPDGINIVQSNGVAAGQDVxHAHVHVVPRYxDDDVx--LxW-SG-DxxExSxQEVAATLRDEL---
>CON.104
x----xxxxCVFCxxVxxKxxAxxVYEDDxVIAFMDxxPVxxGHVLVIPKEHYENIFDIDxxIxxEVxxxVKxVSxALxxxLGxDGINIxQNNGRxANQxVFHYHVHIIPRxxxxxIN----WxRx-xxxxxELExxAxKIRxxxxx-
>CON.105
-------MxSIFTKIIxGEIPxYKVAEDDxFxAFLDIxPxxKGHTLVIPKKEVDYIFDLDDExYxxLxxFAKKVAxAIEKAIPCKRVGVAVI-----GLEVPHAHIHLIPLNxxxDMx--FxxxKL-xLSxEEFxxIAxxIxxxLx--
>CON.106
-------MxTVFTKIIxGEIPGRFVWxDDxxVAFLTIxPLTxGHTLVVPRxEVDxWxDxDxxLxAHLxxVAQxIGxAxxxAFxAxRAGLIIA-----GFEVPHLHIHVFPxxxxxDFD--FxxAxx-xxDxxxLDxAAxRLRxALxx-
>CON.107
-------MASIFTKIIxGELPGHFVWKDDxxVAIMTIxPIRPGHVLVIPREEIDHWDDLPxELxAHLMxVSxKIAKALKxAYPxKRVGMMIA-----GLEVPHTHLHLVPIDxMxDLD--FxxAKx--ADxExLAxxAEKIRxALxx-
>CON.108
-------EDSIFTKIIKGEIPxHxIYEDDxTFAFLDIHPxxPGHxLVVPKxQVDxxxDLDDxxYxxLMxxVKKIAxxLxxxxxxxRVGxxVE-----GFDVPHVHIHLxPFxxxxDxx--xxDxxx-EPDHxxLAxxAERLxxx----
>CON.109 (MccH Hmi, 121 seqeunces)
-------xxCIFCKIVxGxxPxHxIWEDExHLAFLSIFPNTxGFTVVIPKxHxxYVFxxxDExLxxLILAAKKVAxLLDxxFDVxRTGMIFE-----GxGVDHxHxKLFPMHGTxxxI--SSHDxx-RADDxxLxxLAxxIRxxxx--
>CON.110
-------xxCLFCRIAxGEIPAHxVYEDDxILAFLDIxPIRPGHxLIIPKxHYPxFEDLPxDLAxxIMxLGQRLARxMKxLYxVERVGFxFT-----GxxVxHAHAHVVPMxxxxDIT--SxxYxP-xxxxxELxxxAxxLRxxLxx-
>CON.111
-------MSCLFCAISxKExESHIIYEDExCxAFLDIRPINPGHxLVIPKQHxxSLHELxxDxYIxLMKAVRxMSxxVDxTLxPKKVGMAIA-----GFDxDHLHVHIIPMExYHDLT--SQxYIx-xAKEExLxxMKxRLxxx----
>CON.112
-------DDCIFCKIVRGEIPSYKVYEDENFLAFLSIxPINPGHTLVIPKKHxxYxFDLEDxELGELMVVxKKIAxALKKAFNPxKIGVMVA-----GxEVPHAHIHLIPMDxExDLN--Fxx-SK-HxTxEELQxxxxKIKxDL---
>CON.113
-------xxCVFCxIVxGxxxAYxIYEDELSMxILDINPFxxGHCLVIPKRHVPWWHDLxDEExxSLFxVAKxVAxKLxRAFxPDFVAMYAR-----GRRIPHTHIFLVPTxxGD-------------xxD--LxxxAxxLxxx----
>CON.114
-------xDCIFCKIVKGEIPSYKVYEDExFLAFLDIxPxxxGHTLVIPKKHYRWVWDVPN--IGEYFEVVxKIAxxxRKxxxxxxVxxxVx-----GxEVPHAHIxIxPxxxxx-------------xxxxEMxxxxEKIxxxx---
>CON.115
-------xDCIFCKIVKGEIPxxKIYEDExFLAFLDIxPVxxGHxLIIPKKHxxWMQExPDEIIxxIFKxAKKLMxAIKxxxxCDYVQLSIV-----GxDVPHFHIHLIPRYFND-------------xxxxxxxxIxKKIxxxx---
>CON.116
x----xKxxCxFCxIIxGxIxxxxVYEDEKxMAILDxNPAxxGHxLIxPKxHxxILxQIPExLVxHLFxVANKLSxxLFExLKVxGTNILVxNGxAAGQxxPHVMIxIIPRxxxDKIx--xxWxxx-KxxDxEMxxxxxxLxxxxxx-
>CON.117
x----xxxQCxFCxISxGxIQTxKVYEDxEFIAVLDINPANPGHTIIFPKxHIxxxFxLSxxxxEKIFxVAxxLSxxLxNxLxADGxNLYVSNGExAGQKxDHFVIHIIPRFKDDDIS--LxWxPK-KAxQExLKEIxxxLxxxLxK-
>CON.118
------xxDCIFCKIIxGEIPxxKVYEDDxVLAFLDIxPVNxGHTLVIPKxHxxxLxDLPxExxxxLxxxxKKIAxAIxxAxGAxGxNIxxNNGxxAGQxVxHxHFHIIPRxxxDGLx---xWxxx-xYxxxExxxIAxxIRxxLx--
```

```
>CON.119
-----xxxDCIFCKIVxGEIPARIVxETDxxIAFLDAxPLAxGHTLVIPKxHYERIxDMPxDxAxDLFxxVHxLxPxVExAVDADATxVAINNGxxAGQEVPHVHVHIVPRFEGDGGG--AMxxxR-DLSDxELDxIxxxIxxx----
>CON.120
-----xxxDCLFCKIISGEIPSHKVYEDExVYAFLDIYPxSEGHTIVVPKKHFxxFTDMxxEDxAxLFxSVNKIxxxVEKAFSAxGxNIGINNGxVAGQxVPHVHVHIIPRxxGDGGG--SIVxTx---DxxNLxELAExIRxxx---
>CON.121
------xxxCIFCKIVxKEIPNYTVYEDExVLAFLDIHPxAKGHTVVIPKxHxxxLxDMSEExxxxLxxGVxRAxERIxxxLxPDGFNIGINxGxAAGQxVPHVHxHIIPRWEGDGGx--SIIx-N---xxxxVEEVxKxFxx-----
>CON.122
------MxxCLFCKIxxxEIxSxxIYEDExTxAFLDIxPRAPGHTMVIPKKHxxxIxDLxxExIxxLFxxVKKVxxxLxKxLxxDxFTIGINHGExAGQxVxHLHVHIIPRFxxDGGG--SVVx-N---PKESLxxIxEKIxxxx---
>CON.123
--------MCIFCKIVNKEIPAxIVYEDDxxLAFLDINPxSKGHTLVIPKxHYExFDELPxExLxxLxxxIKKVVExLKK-LNxDGYNIINNNGxxAGQEVxHVHFHIIPRYExExxx---xxxxx-KIDLDEIxxxIxx--------
>CON.124
-------xxFxLxPRL--xxDTxxIGDFPLCRLLLxNDxxYP-WxILVPRRxIxEIYQLxxxDQxQLxxESxxLAxxLxxxFxxDKMNVAAL-----GNMVPQLHIHHIARFxxDxAWPxPVWGxxPxYxxxxxxxxxxLxxxLxxx
>CON.125
------xxxFxLDPRL--xADSxxVxDxPLCQVRLxDDARFP-WLILVPRxxxxEIxDLxxxDQAxLxxEIxxAxxALRxxxxxDKLNIGAL-----GNVVxQLHVHVVGRRxGDAAWPGPVWGxGxxYxxxxxxxxIAxLRxxLxxx
>CON.126
------MxxFxLDxRL--xRDSxxIxxLGLCELRLMNDxRWP-WLILVPQRxVxEIFDLTPLDQxMLTFETNxVAxALKxVTGxxKINVGAL-----GNIVRQLHVHVIARxEGDxNWPGPVWGxGxPYxxxxxxxFxxxIxxALx--
>CON.127
------MxxFxLNPRL--EGDSLxVADLPLCxVRLMKDANYP-WLLLIPRxxLIEIIDLSExDQxQLMREIAxASRALRxVTxCEKLNVGAL-----GNQVSQLHVHVIARFxxDAAWPGPVWGAxPPYEPxxxExLIxxLRxALAxx
>CON.128
------xxxFxLDPRL--AADTIPVGDLxLCSVLLMDDARFP-WLILVPRRPxxEITDLxxxDAxxLxxEIRIATxVMxxLAKPDKVNVGAL-----GNVVAQLHVHVIGRFRSDPAWPGPVWGxGTPYPxHAxAQLIERxxALFAAA
>CON.129
------xxxFxLDxRL--xADTxxVxDLxLCRLLLMNDxRWP-WLILVPRRxIVELFDLxxxDRAxLxxEAxxVARALKxxxxAEKMNIATL-----GNVVRQFHLHVVARxxxDPNWPxPVWGFxxPYxPxEAEAxxxRLxExLx--
>CON.130
------xxxWxLHPQL--AxDTHPVxxxxLxEVxLMDDANYP-WLILVPRxxxxExxDLDxxDQxxLxxEIxxxSRALRxxFxPxKLNVAAL-----GNMVPQLHVHVIARFxxDxAWPxPVWGxxxPYxPExLxxRIxxLRxxLxxx
>CON.131
--------MFxLHPRL--xADTVxVxxWPLCRVLLMNDRxWP-WLILVPRRxVxEIHxLxxxDQxxLMxEIAxASxxLExxxxPDKINVGAL-----GNMVPQLHVHVIARxRDDPAWPGPVWGxGxPYxxxELExxVxRLRxxLxxx
>CON.132
-------xxFxLHPRL--xADTLxVxDxxxCRVLLMNDxRYP-WLILVPRxxLRDFHDLxxxxxxxFMEEIRxVSEVLxExxxAxKMNVAAL-----GNMVPQLHIHVIARFExDAAWPGPVWGVGxPYxxxxxxALxxKLxxAIxxx
>CON.133
-------MxFxLDKRL--EKDSxLVxxxxxFQIRLMNDxRFF-WIILVPxxxLTELHDLxxxxxNxLxxxAxxLGxxLKxxxKxDKINIGxL-----GNIVSQLHLHIVxRHxxDAAWPGPVWGxGxxLDxxxKxxRxxLIxKxLxxx
>CON.134
------xxxFxLDxRL--AxDTLxLASLxLCQVLLFNDxRYD-WLVLVPRxxCxEILDLSxxQQxQLWREVxxVAQxLRxxQPxxKLNIGAL-----GNVVxQLHVHIVLRxxGDPAWPGPVWGHSPPYxxExxxxAxxxWQxxLxxx
>CON.135
--------MAxLHPRL--AADTxALGETDLCWLRWMNDQRFx-WLIVVPKRDLREWHHLPxxEQQALLxxVNxLAxELERITGADKINIGAL-----GNMVPQLHIHIIARFxGDPCWPGPVWGQGxPWxxxQxPxWLxxLxLxxxxx
>CON.136
------xxxWELHPQL--xADSFPVxELxICQLRLINDQRFx-WLLLVPRIAAxELxDLxPxxxQQLxxEIxxVSRVLQxxxQPxKINVAAL-----GNxVPQLHIHCIARFxDDAAWPxPVWGxGxPYNPxDxxAxxNxLxSxxAxx
>CON.137
--------xFxLHxxL--xxxSxxIxDLxLCxIRLxxNxxFP-WIILIPKRxIxEIxDLxxxDQxxLMKEIxxxSKxMxxxFxxxKLNVxxI-----GNxVPQLHIHVIARxxxDxAWPxxVWxxxxxYxxxxLxxxIxxIKxxFxxx
>CON.138
------MxxFxLHPQL--xADCHxLGxLxxxxLLLHRNAxVx-WFILVPETDAxDLLDLPxxxRxxLLxxCxxISxFLKxxLGxPKVNIAAI-----GNLVPQLHLHVIGRRPxDPCWPxPVWGHLxxYxxxEIxxLxxxLxxxxxx
>CON.139
--------xFxLDxRL--ExDCFEIxExxxxxxLLMNNxxIP-WFIIVPxTDxxELYxLxxxEQxxLxxxINxISxFIxxxFxxDKLNVAxI-----GNVVKQMHIHIIGRxExDPxWPxPVWGNxxxYxxxExxxxxxxLxxxxxxx
>CON.140
------xMDxIIPPEx--LFESxWLYEHPLYSLMx-EKTEIP-WLLxVPKQxLxxx-xxxYxx--xLYGxIYQLxDxLQxxGLGxHFNIAKI-----GNKxPYxHIHLVFREPNDExWPDxIWCHExxSx-ExPEKLKExLxxFYSxx
>CON.141
-------MxFxIDxRI--xSSSxxLxDWPLSRVYLKNDAxFP-WxILVPRxEIQEIYQLSxxxRxxLMEEIxxLSxIMxxYFKPDKLNIGAL-----GNIVSQLHIHVVARFxxDxxWPHGIWQxxxxYxxxxLxxLVxxLRxxIxxx
>CON.142
------xxxDWRxDRIG-GENPTVLxRLxxGFAVIGDVQFLPGYCVLLxxxxVxxLxDLPxxERxxFLxDMxxLGEAVxxACxxRRVNYEIL-----GNTDxFLHAHVWPRYxWEPxExxPVWLYPxxLGP-RHDxLRxxIxxxLxxL
>CON.143
--------xxLIxQRVx-GxNPxVIxRMxSGWAVMGDVQxLPGYCLLLPDPVVPSLNDLxxEARxxYLxDMAxIGDALLxATGAxRINYEIx-----GNxEPxLHxHIFPRYASEPExxxPxWxYD-xFxxxxHGxLxxxIRxxLxxx
```

>CON.144
--------xCLICERIx-GxNPYFVKELETGYVVIGDHQYFKGYTLFLCKEHxTELHxLExxxRxKFLxEMSLVAEAVxxAFxAEKMNYELL-----GNGDAHLHWHLFPRxxGDxxxKGPVWWxPxxPxxxELExMKxxLxxELxKL
>CON.145
------xxxCxxCxxVx-xxxxxxVWxFPxSVAFLGPxQxxxGYCVLxNRxHASELxQLGx-DRTAFLDDMAxLAEAIxxCFxPHKMNYELL-----GNxVPHLHWHIFPRQxxDPxRLHPVWxAxxRLGxxPxxEVVERLRAWLTxN
>CON.146
---W--xxxCGMCxDAH-NEHSxLIxxTxxSYVRLxRNQxHxGWxIVFLREHVxDLxELxxxQLSxFWxDVQRAARAVxKVFxPxKINYLVM-----GHRxPHLHCHIFPQHxxDDPxxNVx--IxDxLxDDEYDxxVxALRKAWxxS
>CON.147
------xxxCxYCxxxE-xxLMIxIxELxxSxLYLFKDQTHxGRCIVAxKxHxxELxELSxEERxxFMxDVxxVAxAIxKxFxPDKINYGAY-----GDxxxHLHFHLVPKYKDGxxWGxxF-xMNPxLxDxEYxxMIxxIKxxLx--
>CON.148
-----------------EKFGxxIKEFDxSxxYLxRDQTxRGHCVVVxKESxSxYxRLSTLERNTFxADIxRVxKAIxKxFxPDxIQYIYx-----xDVDxQLxFHIIPKYKDxETYGKxx-xxDxxLxEEEYExLxxQIxENLx--
>CON.149
----xx-xxCxxCGFxL----WxPIAxLSVSRVGLYDDARFPGRxIVSLREHYDHLDEAPxxILxxFMADIQxASxVLRRxxGVERVNIAIL-----GNxxxHVHAHVIPRRxxDxNxGxAPWDxAxxLxPxxRxxLVxxLRxxFxxx
>CON.150
----xxGxxCxMCxxxx-xxWGIRIxxGEVADAYLxRxGxxRGYxVVIWRGRVxEPTELSDxEAAAYWxDVLxVGRALxxxYxPLKMNYxxL-----GNxVPHLHTHVLPRYxDDPAPGxPLxxxLDxxDxxQLxxDAAALRxLLxxx
>CON.151
------xxxCPMCSKWx-DDPxLRIAELEHxxVMLNRDQFFPGYxFVFTRxHVTELFHLDxxVRxGVMEEVSAVAAALxxLFQPxKINYELL-----GNMVPHMHWHLVPRFATDPLWPRPIWSEPHxLxxEEYxERIELIRxxLxxx
>CON.152
------DxxCKACxGTW-PRxDHFIADLGLSRAYLHDDQFFPGWTVLVFQRHATELFHLSPxERxxLIEEVxRVAxxLAExxxAxKINYELL-----GNQLPHIHWHLIPRLADDPAPLEPVWRVPHxLxGxxLxxxIDRLRxxxxxx
>CON.153
------xxxCxLCxxxG----GxIIWRDxxxRVVxVDDxxYPGFCRVIWNxHVxEMSDLxxxDRxxLMxxVxxVExALRxxMxPxKINLASL-----GNMVPHLHWHVIPRFxxDxHFPxPVWAxxQxxxxxxxxxLxxxLxxxLxxx
>CON.154
------xxxCPICxAxx----EDILxQxxxLRVIAVDExxAPAFCRVIWQEHVAEMTDLxPAERxELMExVYRVEAAMRQVFxPxKINLASL-----GNVVPHLHWHVIARFEDDAxFPAPIWAxPxLPE-DWxxQVxxLLxxxx---
>CON.155
------xPxCPLCQxxG----GxLLWRGxxLRVIxVxDxDYPGFTRVIWNxHIxEMTDLSxxxRxxLMxAVYxVExVQRxxLxPDKINLAQF-----GNMVPHLHWHIIPRWRxDxHFPDAIWAxPRxxxxxxLxxYxxxLxxxLxAL
>CON.156
-------xxCPLCAxxx----ExxIWRNxxFxLIDVSDPxFPxYFRLIxxDHVxEMSDLSxExRxxMWDLLxxLEEAMxEAMRPDKVNWAQF-----GNMVPHLHWHLIARWxDDxxFPECPWxPxQVIxExxxExLxxRLAxxLxxx
>CON.157
----------------------MIYENxxIxI-ExExSEIP-WIKIFTKxxYKELSDCxxxxRxxLFExLxIxEKxMLxYYxPxKINIASF-----GNYVPRVHxHVMARFEEDSFFPExMWGxKQLPx---FExFxxxLxxxLxxx
>CON.158
-------xxNATxxKF--GYPxTLIxEYxHWxVLLRPxQPTLGSLVLVxKExAxAFGxLxxxAFAELxxVVxxIExxLxxxVxYEKINYLML-----MMVDPxVHFHVIPRYxGxRxFxDAGWPGxPxLDxxxxxxLxxxLxxxWxx-
>CON.159
------xxLxxFRxKF--RVDELLIxxxEAWxWSVRPxQxTLGSxILSLNRxAxxLxEVTxxExAxLAxIVxxLExxLxxxFxxDxINYLxL-----MMVDxHVHFHVIPRYxxxRxWxDxGWPxxPxxxxxxLxxLRxxLxxxxxx
>CON.160
-------xxx---xxx--xYxxxxIKEYxYWTIxIHxNQxYLGRCIIWxKRxx-DLSDxTxEExxELxxVLxELxxxxxKLFQPDWFNYxxL-----GNxxxHxHxHFIPRYxxxRTFxDxRWGHNYxxxxxILxxIRDxIKxxLx--
>CON.161
---xLxxxxxxxGxxM--DYNQLKIKSFKHWDIYLHENQCYLGRVFVQLKDGIEDFLDIDxEVRDEFFQIGQNVKKALKxLFKPDKMNYAAL-----SNTSPVIHMHIIPRYKEPRTFKDTRWGQNYVIDExTLFKIRDALKxQL---
>CON.162
-------xxCxxCxx---xxxGxxIxETxxWxVFLAPxQxxLGTCVVALKRHxxxLSxLxxEEWxDFxxIVKKLExALKxxFxATLFNWxCL-----MNxxPHIHWHFIPRYxxxVxFxDPxFGxxxxIxxExxxxIxxxIKxxLxx-
>CON.163
---ELxKGICxSCYNxx-E--GxIIYEDDKVRCxFEKxPRATGHTxIxSKEHYEDISEMPxELGxHIFxISxxIIxLxKEIxGAEKV-YMCT-----MCDRNHFHFQLFPRLKxEKGxKNF--VKEExETxxLYKEKLKELxx-----
>CON.164
-----------------xSPKAMIYEDQxLYVCLAxxPITRGHTIVAWKExVPDLNxLxERDYDYLMDTVNAVRxAMxKVLxIKKV-YLxY-----MDEARHVHWHLVPRYx-EKGFxVF--LHQPSLAPxI-xRxL---KxK----
>CON.165
--------DCxxCxxxx---xxxxIWxNERWRLxxxxxxGLPLVLMLxPxEHx-DLxDLPDELAAExGxLxxxIxRxVEALPxVGRxHVxRx-----GDGGAHLHVWFFARPxxxxQLxGSxWD-DLxxPxDVxxADAxxVAxxLAxx
>CON.166
PG--x-xxDCxACx-AP---DDAYIWVxERWRVRAMRPTGLPMVLILExRSHL-DLGDLPNLLAAELGVMTVRLERAIRSLDGVARVHVNRW-----GDGSAHLHMWFLARPYGRLQLRGTLWD-DIPIPExQWRENLALVAAWLADx
>CON.167
-------xxCxxCx-xx---xxxxIWxDEHWRLxxxxPxxLPxVVxLxPRxHx-DLxDLPxExxAELGPMLQRVERAIxSLGGVARVHVxRW-----GDGxAHLHLWxxARPxGMMQxRGxLWE-DVxLPxxxxxxxxRxIAxAMAxx
>CON.168
--------DCxxCxxAA-xDxxYVVWRDDLxMLGxPExSLPFVxFLMPRRHA-DLSDLTPxEAARMGELLVxIERAAxxVLDVPRIQVxRW-----GDGxEHLHWWLYARPTGMxQLRGTxWD-DLAxPxAExRADxExVAxRLExA

```
>CON.169
----P-xxDCLMCRGxD-ADPxxQVWEDxxWRTA--xxGEAPGFSxLEPxRHIxYVxDLDGxEAATFGxVLARSTxAIREATGADLVYVYVF-----GGxFDHLHLHLAPHxxxGPFDLGGxxxE--VLxxGAVQAAAEAIxxALxR-
>CON.170
-x--V-NDPCLGCAIVx-GxVGGVLARAGLVLHGVAGPSPIPGWVVLTSERHARAWYDLDxxxARELGPFAARVMRAQREVLGAEHAYAFAI-----GDVLRHFHLHLVPRYxxTPQLWGRDA---xHLPxxxLEAAARALAAALxx-
>CON.171
-x--x-xxxCxICxLHx-DxxYE-IxRxxxWVLRHPxPAPLxGWxLLDSxRHxSGPIDFxxxEAxxWGxAVQxASxLVKxLTGCDRVYAIAF-----GEGAQHLHLHLIPRFxxDPx-xAWDxYRAVAAxxxxVxxFVxxARxxxxx-
>CON.172
------xxDCPICxxHR-xxxSEILFQxxxWxIRHAxxKKLxGYLYIExRSHxESWxDWxxxEFEDFGKALxxATEWIxKxHxPxKIYTVxI-----AExVPHMHFHLIPRYExDx--KGIDYIRxAxxPxPxxIxxx---------
>CON.173
----S---xCLxCxLEx-ADDxAVVFRDxLWAVV--PGYDVPGWFVLRxRRHAERxTGLDxxELxxFGRRARDLVAAVTExTGAPATYxMxF-----GENHPHFHxLVxARGxDVPxRRxGxILxLRHADPxAAxxLVPxVRxAYxRx
>CON.174
----T--TxCxICGMxx-ATDxDIVYxDxxWTVA--SxxPVPGWLMLFTRxHNRGxWxLSExAAAQLGPLIxxLTGALxxTxxxERVxVMYx-----GDNALHxHFMLMGRGPDxPxWxGAxLIxxHxxDxDRARxVLADLRExLxxx
>CON.175
----x-xxxCxxCxxxx-xxPGGWIxxDEHWxVxHGxxxxxAGTLxIxSRRHxLDFADMTxxEAASFGxLLxRLxxAVRxATGAERVYxxST-----MDxxxHFHxWLVPRxxxxxx-RGxxxLx-xxxxPxEAxxxAxxIRxxLxxx
>CON.176
----x-xxxCYxCxxxA-xxPRExIxxDxHWRVAHAFNTALPGWLVLIPRRHVTSIAELTxAEAAxLGxWxxRLSRALxxVTGCxKTYVxQF-----AEGFxHVHFHIVPRxxDLPxxRGPxxLxxxxVxxxxxDELAxxLRAxLxxx
>CON.177
---------CxACxLxx-GxPGGxIxxTxxWVVEHCVGPLGLGTLVVKPxRHVVHIADLxxxEAxELGPLLRRVSxAVxxIxRPEQVYVCLW-----Sxxx-PxHIHFQPVxxxxxxYxGPMxxxGxx---xEVExFCxRxRxxLxxx
>CON.178
-------------------MPxFIIHETxHWLLNHHMxSALPGYLMLGSKxxxNSLADLPxxALAELGxLLARVQxxLExxLxPKxLYIGRY-----GPGY-PIHFHFIPVYDWVLQFGGAEFCERPxIQGPSVxQVIxxLRxAFxxx
>CON.179
--------------------DExxIFENxHWxVSHRRDARYxGYLIISSxxxASDLxDLSxxALASLGxVLxxxExLLxxxYSPYKVVFYKL-----GxGF-NxHFHVAPVTQx-LxIxGNEYCERxxExxKQxx-xVxRLRxxxxxx
>CON.180
xx--V-EVECLSCAITS-GxxGGVIxETEYF-HAHQVAYPIxGLVILASKRHIxxLDELTEEExxDYIxLLxKIRKAQRxxLGIExVYYFYN-----EDTTHHFHxWMVPRYEWMxx-FGR-SVRPVEENxxxVxEAIxxLRxxLxxx
>CON.181
xN--x-xxECIGCAIxK-GDPGGIIYDxKNF-ILxAPEVPIPGFLIITxKRHIRSFAELTxEExxELGDVIxKAExAIKxLxxxxEVTLVQE-----E-RSxHFHIWIFPxYDWMxK-FGx-xIRDIExDxEEVxxTAExIRExFxxx
>CON.182
--PYPxExTCRGCFMNxAELPDAPVYDDGxITVRQDAEWAVPGFMVVGVRPHIGSLDEMELxLxxRLARITHFVRRAMRERLGLxAVQMYQE--E--KLDRPHFHxWMLPLWPDTMAA--HxI--NPRIYExxIRRCxDEVRDFLxEx
>CON.183
----x-xxDCFICxKHx-GxxGxxIYEDDYVYVGHIGxPxYLGHIMIDLKRHVPxLGDMTxEEAKAFGLIMARVSKALxExEGAEHIYSxVS-----GNSVPHLHMHLVPRYPNTPKYWGxDW---xxGxxxEVxxLCxRIKxYLExx
>CON.184
-------xxCxICxKHR-xxVGPVVWADDLVVVTHRxxxxxPGYLFVETRRHVxxLxxLTxxEAxAVxRAVxxAARALRAELxPExVFSAIA-----GRSVAHFHQHVFVRPxGTPxVxWxSW---xRIDxxxLxxLCxRLAxYFxx-
>CON.185
YR--R-xGPCFICxLVA-GxxHxVVYEDxxxIAFLxRYPTLxGxxLVAPKxHxEHVxDxxExxYLRLQxxVHRVAxAVExVxPxERxYILSL-----GQGNAHLHWHIAPLPPGVPYEQQFMxExGV-xxxxExAxLAxRLRxALxxx
>CON.186
TMxHxxxGxxLFxDILxxExGxRVVxxxEHWTAFVPxAARWPxEVHLxPxRxVPDLxxLxDxERxEFAxIYLDLLRRxDxLFxxxPYIAAWHQAPxxxRxxxxLHLQLFSxLKYLAGS--E-SGMGAWINDVxPExxAxRLREVxxx-
>CON.187
xxx-------WRPD-----xxDRLVxxxxxWxAxVPxAPVYPVAIxIAPxxxIxxLxxLSxxxRxxLAxILxDVLxRLDRLFxxLPYMMWIxQxPxxxxxxAWMxIEIVSPxRYIAAA--E-VASGEYFNPVxPExLAERLRxLx---
>CON.188
xExYxxHGxxLLxDYxxxExxERIVxENEHWVAVVPYWAxWPFETMLLPKxxVxxLxELxxxQRxxLAxxLKxLTxRYDNLFxxFPYSMGWHxAPxDxxxHWxLHAHFYPPRKFMVGY--E-MLAE-xQRDITPEQAAxRLRxxxxx-
>CON.189
xxxxxxxxxxLLxDxIxxExxxxIIxxDxxAIAFVPxFARYPYEVWIxPxxxxxxxxDLSxxERxxLARxLKxVxxRYDxLxxxMPYLMAxHxAPxDxxPxxHxHVEFYPxLKYLAGT--E-xxAGxFAxDxLPExxAxELRxVxxx-
>CON.190
IEAYRETGRxLFxDIIxxEDGRRIIxENExAIAFVPYFARYAYEVYVxPKRxxPxIxxMxDxEVxxFAxxLREVxVRYDNLWQMFPYVMxLHQAPTDDYRxFHFHIxFHPPxKYLAGP--E-IGGGNFxSDTSPEEKAAELRAxxxx-
>CON.191
xEAHxRTGRCLxCDVLAxEDGRRIVxENxxFxAxVPFYARYPYEVHIxARxHxxSLxDxxxEExxDLAxILKxVLxxYDxLFGFLPYIMAxHQxPTDxxxxxHFHIEFYPPLKYLAGS--E-xGAGTFINDxxPEExAxELRxxxxx-
>CON.192
IESYxExGxCLICDMxxEExxKRIIxENDxFIAFIPFFTDYPYGVYIxxKRHxxxLxDFTxxEKxDLAxILKxVTGxxDxLFDxFPYMMxxHQxPVNxxDYYHFHIEFYPPIKWxASS--E-TGAWAxxNPxxxEExAxxLRxAxxx-
>CON.193
VENYxxxGxCLFCxILxxExxxRVIYxNxxWxxFLPFYAxWPYEVHIYPKRHVQxLTELxxEEIxxLADxLRxxLxxLNxLFxxMPYIMxLHQAPxxxxxxYHLHIEFYxxLKYAAGI--E-xGxxxFTYDxVPEExAxxLREAxxR-
```

```
>CON.194
IESYNEKNSCYWCDxxKxxAxTRIVYESxHFVLxVPxACRWSYEMILIPKNHKPNFGFMDEMEINDFAxVLxGALRAYDxLFDRPDRNFWIHTM---RYEPYHWHVGFIPHIKVFGGL--E-LGAGIWVSDATPEDAAKQLSxxVKxx
>CON.195
VEGYxxKERCIFCDIIxQExxxRIVxENxxFIAIxPFAxRFPFExWILPKxHxxxFxxxxxxxLxxLAxILKxxLxRLxxxLxxPPYNFVLHTAPxxxxxxYHWHIEIIPRLTRVAGF--E-WGTGFYINPxxPEEAAxYLRExxx--
>CON.196
VEGYxxxGxCxYCDMIxxExxxRVVxENxxFIAFxPFASRFPFETWIIPKEHQxxFxxIxExxIxxLAxILRxxLxRLxxxLxxPPYNLVLHTAPVxDxxxYHWHIEILPRLTxxAGF--E-LGTGxYINPTPPExAAxxLRExxxx-
>CON.197
IREYDxxGxCVYCDMLxxExxERIVxENxxFVAFVPYAAxxPFExWIxPRxHxAxFGxIxxxExxxLAxVLRxVLxRLYxxLNNPDYNYVIxSAPxxxxxYxHWYIxIxPRLTxxAGF--E-LGSGIxINxSLPExxAxFLRxxxx--
>CON.198
VExYxKxGxCIFCSLIDEAVGQYIIERGEHFVAIKPFASRYEWEVHILPLKHxADFxxVxEEEMxDLAxVLRRTMARLxxVLGGxQYNFFLHSVPHxxxxSYHWHLEICPRTSIPTGF--E-LGSGLFVNTVSPExAAEQLRxVxI--
>CON.199
VExYxxxGxCxxCDIIEKExxxRVVxxSxHFVVLAPFASRxPYEIxILPxxHxPxFxxxxxxxxxDLAxxLRRVLxxLxxxxGDPxYNxxLHTxPxRxxxxYHWHLEIxPRxTxxAGF--E-WGSGxxINxxxPExAAxxLRxxxxx-
>CON.200
xxxHDxxGRxxxxDxxxxEDGxRVVxAxDHxVAFAPFAxxxxYETWIVPRxxxADFAxxPDxxLxxxAxxLRRVLxALRxxLGDPxYNYVLxTAPxGxxxYxxWxLQIVPRLxVxAGF--E-LGTGIxVxxTxPExAAAxLRxxIxP-
>CON.201
VERFRTMGGNLLADLVQEExRERIVAIDDExVxMAPYAARxPFHLMLAPRRPxARFEDxDGxxGA---ALLHDALxRLRxxLGxxPLNLWVRTAPxG-AEHFCWRIDIxPRLTHLAGL--E-LGTGVxLNIVxPEQAAAxLRxx----
>CON.202
MEAFRFxGGCLLCTTIEAExGERIVxxNDxxVVICPYWSGxPYEMLIIPRxHxxHLxxASxxxLxxVGxxLRDALxxLxxxLGDVAYNLxFHTAPHQ-HGxFHWHVHIWPKLTTxAGF--E-xGTGVxINIVxPExAAxxLRxxxxx-
>CON.203
VSGYKKxGQKIFDVLLDWEDxKRIIYENDxFVAFCPYVSKxPYEIRIFPKxDHAxFxxLxxxDxxxLAEALNxxLxKLxxALDxPDYNFFIHTAPIRxHDYYRWHIEIVPRLSxxGGV--E-LGTxVYVNVVDPDDAAExLRxxx---
>CON.204
IKxHExKERSLFADVLxEESGxRIVYENSxFAAFCPWASRFPFExWIxPKxxSxDFHxLDDxQLxLLADVLQxVLRRLKKGLxNPxYNMILQTAPxRVExDFRWHIEILPRxxxxAGF--E-xGTxFxINxVxPEEAAxFLRxIRxx-
>CON.205
LExHExxGxSLxxEIIEQExxxRVVxxTDHFVAICPYASRFPFETWIIPxRxxSHFxxLxDxELDDLAxIxQRLLxxLExxLxxPxYNYxIHTxPFDExxxYHWxLEILPRLTxxAGF--E-WGTGxFINPVPPExAAAxLRxxxxx-
>CON.206
VVRHARxGxCLQCDIIRxEEKQRIVxQSDSLVAYCPxASRxPMxVRITSKDHxxxFDELxPxxLxELARxILRxVRWLExLxPxTAYNMMLHTxPxGNxEAHHWSIEIxPRISRLAGF--E-LATGxMINTIxPExAAxEYRxxAxxV
>CON.207
--xYQxNxRCLMCDxLEQExxERIVxESDHFIILCPYASRFSYETWIVPKRHxEHFGDIxEEEIxDLAxIxKxLLxAMxDxLxNPSYNIVFNTAPVNxEPGxHWYMEIxPRLIVxxGI--E-IGTGYYMNPVxPExAAxxLRExLxE-
>CON.208
VxQYxEKxRCLLCDxIxxExxKRVIxENExFIAYCPYASxxPFEMxIxPKxHxxDFxxIxDxELxxLAxVLxxxxRKLxKxLxNPPFNMxLHTxPxxxxxFFHWHIEILPRIxxxAGF--E-LGTGxYINPTxPEEAAKxLxExxx--
>CON.209
VSGYxxxKKCVHCxMIExExKKRIIFENExFIAFxPFASRxxFEVRIFPKKHxPYFExIxxEExxxLAEALxxxLxKIxKxLNDPxYNFFIHTAPxDxYxHYHWHIEILPKTSxWAGF--E-LGTGIEIxxIxPExAAxxLRKx----
>CON.210
IExxxxxxxCPYCxIIxKExSxRxIxENxxFVAFAPYASxxPYExWILPKRHIxNLxxLxxxxxxSLAxxLRxVLxxLxxLxxxxPYNYxFxQxxxx--xxYHFHLRIxPRxSxxAGF--E-LNTGIYINTVxPExAAxYxRxxx---
>CON.211
IEAxNEKGxCPMCQVIxxExGPRQILQTDGFIAFCPWAPxYPYEFWIxPKKHxTSFSKISQKEINDLALILRATLGGLxxxIKDxSYNFxFHLSPEKNSRQIHWHIEVYPxTxxWSGL--E-RGYGIFLNxISPExAAxxLRKELAN-
>CON.212
ID-----------xxxxEPxxNxVxExxxFxVYCPxFSQWPYEVWIAPKxxGxxFGDITDEEIxDLAxILxxxIxxLxxxHxxFxYNFYIYxx-------xDWYLRIIPRxxHRGGF--E-LGTGIxVNxxDPKxAxExLxx--xx-
>CON.213
IxxYxxNxxCxxCDxIKxExKxRIVYxGKxFIAFCPYASIFxYEIxIVxxxHxSSLLNLNEEEIxxLSxIIxxVxNKLxxxLGDIxYNIxFxFIKE-ENEYYHFYIxIxPRxxxxAGF--E-ISTGIMxNxVxPExAAxxLRxxxx--
>CON.214
xMx--QxxDCLFCKMLAYEQKxRIVxENEHFLAITPYASRFPYELWIxPKxHxxSFGxLxAEELxDLAxLLxxMLPRVTxLREDVGYNICLMDGxxxD---FHWHIEILPRIGGFAGF--E-FATDxYINSVxPExAAxYYRKEx---
>CON.215
xxRYxxHGRSLxExIIxxExKxRIIxxxxxFxAFCPYASxFAFEVMIxSKxxxxxIxxLSDxxLxELAxLLxxVLxxLxKQLGxFDFNLxFxxPPLNxxExxRFxIRIxPRLYxLGGF--E-IxSGIxINPVxPExAAKLLRxxxxx-
>CON.216
IEVYxxxxxNLYExIIExExxERVVYNGExFLVxVPxAxxYSGEVRIIx-KxxxKFExLxDxxIxELSxIFKKLFxKxxxxxGxxPFNLxIHTHPxxxxDxFNxHIHIIPRKFNFGGF--E-LSTGMYVSSVxPEELAxKLRFx----
>CON.217
IExYxxxxxCVYCDIIExExxERIIxENExFIAIxPFASxYxYQxYILPKxHxxSFxxxxExEIxxLAxxLKxVFNRLxxxLGDFxFNMYLHTLxKxxxxSYHWxLDIxPRMSxxAGF--E-LGSGVxINSVxPEDAAxxLRxxxx--
>CON.218
xER----NPNLYNExVDYAxxNLVIAENDHAVAFAGFGHRYPT-LEVWSxSxxxxPWxxSxEEIRxMSDLIHAxHAA---TGxxVPCNEEWHHRPxDVDxPMPWRIxLKWRVSTLAGF--E-GGTKIYINTIDPWxLRDRVVxxLxxx
```

>CON.219
xxAYxExGxxYWxDLVxxExGERYLxQxGxxxWLxxFAPRGLxDVxAIF-xGxxTxxDLxxxDLxELAxGLxxVLxYYDxxN-IxSFNxALYxA--xEDxGFxxHxRIxxRxSDISxL--Q-ILxxDxxxIxxPExIAxELKxxFx--
>CON.220
FEAFxxxxxxFWxELIxxExGERYIGQIGDxHWLxxFxPLGMGEIMCVF-PGxxxIxDFxDxxxxxLVxGLxxIFxYFxxKG-IYSFNASLFFG--PGQxxFPAHFRIVPRAPDLNFF--Q-AxLQEPVxVVxPEDLCREIRPYFxx-
>CON.221
QIxFxxxGKEYFTxLYxTExGERWIGExGNVAWMHAYAPKGHNDFIGIF-xxxxSFxDIxExDWDDFAxGLxxIFxxFKEQG-FxSFNLxLxIS--VxNxxQxVHVRLIPRTSDINxF--Q-xLHQEPLSYKxPEEVAxxARKFFxK-
>CON.222
HLRHxExGxRLFSDYLxxExGxRYIGATGxWEWLAAFAPEGFYEIWGIL-PGxTSLxxPxDxxWQxLARGVINVQKFYRSLG-RNGYNLGLLxLEDxNDLELR--VVLTVRRSDFTGF--E-IMLGDMATFTxPExTAExARxFWQx-
>CON.223
QLxYExxGRxYWxELxExExGERYVGxxGxxxWFxPFAPxGFYHVxGVx-xxxxDIxELSDEDLxxLAxGIxxVLxxYxxxG-LNSFNFxLxGAxPxxxxxxxxLxRIVARxxDVTYF--E-KLHxEAMVDxxPEExAxxLRxxF---
>CON.224
HLKYxKHGKNFWEDLCxxERGERWIGQxGRxCWITSFAPMGxNEILAVW-PGRQGFLEWTxQDVWDLARGLNxVLKxWHEMG-xGTFNFTCFxPPLxDSPHFCxxMRLVTRRTDDYFL--Q-RxMxNELIITxPExMAQxMRExFxx-
>CON.225
--ALNKRHxKSFSQYMLEENPxLxVKDYGEVxLVVPYFMKRPLDMLLVKDSExRYLHQLSxxEQQQVVxGIQxAIxAILQMGxPPAYNMIVNNGPGCG-----LYLEFLAKTQxMGGY--EQIGL--FVCQANAxDSAxxLRQxIxx-
>CON.226
AEIFE--xGCHLCTSR---HDDRIIYENETWIxWATSSPRRNYHVRxAPKRHVxRFTDLxxxEIxGLAETLKxVSxAMDKIGIxRDRNVLFYSxPYGYDSFFHLFVDIIPFEx-IGGI--E-MLDSVRVARVxPEEAAKxLREALEK-
>CON.227
-x---xxxxCPFCxxPxxDEDGLIVARGExVYVVLNLYPYNPGHLMVCPYRHVADYxDLTxxExxELxxFTQxAMRVLRxVSxPxGFNIGMNxGxVAGAGIAHLHQHVVPRWxGDANP--V-IGxT-KVLPQLLxDTRxLLAxAWxxx
>CON.228
-----DxDGCxFCxLPERDRENxIVARSDHAFVLLNNYPYNPGHVMVIPxxHTGDYxxLxDxxLLDHARLKQRTxDALExALxPDGFNAGLNLGxGAGGSIDHLHTHVVPRWxGDTNP--V-IxDT-KVIVEALDDTYDRLHEAFAxD
>CON.229
-------xGCVFCAxAAADAENLVLYRGQxCFVVMNLYPYNTGHLMVVPYxHTADLxGLxxETAxELFxLTQRSVAILQTxYxPHGFNLGMNLGRVAGAGIxHLHMHIVPRWNGDTNP--I-IGGT-KLIPEALDDTYARLRPxFxxx
>CON.230
-------xxCIFCxxxxxDxExLILYRGxxxFVIMNxYPYNNGHLMVxPxRHVxxLxxLxxEExxELxxLxxxxxxVLRxxxxPxGFNIGINLGRxAGAGIxHLHxHIVPRWxGDTNP--V-IxDT-RVIPExLxxTYxxLxxxFxxx
>CON.231
-----xxxxSVFxxILxSDxETFIVHRGxxxFVILNAFPYSSGHLLVLPxRxVAxLxDLTxDExxELWxxVTDAVxALKxxYxPEGLNVGINLGxAAGGSVxHLHVHVVPRWxGDSNT--A-VAxT-RVLPEALxxTxxRIRxxWxxx
>CON.232
------xxxxxFxEIxxxDxxxLILxRxRxSFxIMNRYPYNxGHLMVVPYREVxDLxxLTxxExxDLxxxIxxxxxxLxxAMKPxGFNIGFNLGxAAGAGIxHLHxHIVPRWxGDTNP--V-IGxT-RVLPQxLxxLWExLxxxxxxx
>CON.233
-------xPSIFxxLAAEDEDNLILWRGEHVFVIMNxYPYNNGHLLIVPYREVAxYDALTxxEQxxMTxAIxRCIRWLRxALKPDGFNVGMNLGRAAGAGIPHLHxHVVPRWxGDTNP--T-TAET-KVLPExLRDTYRKLRAAIDAD
>CON.234
-------xGCxFCxAxxxDxExLILYRGxxAxVILNRYPYNxGHLMVxPxRHVGxLxELxxxExxELxxLxxxAVRxLxxxxxPxGFNIGLNLGRxAGAGIxHLHxHIVPRWxGDTNP--V-LxET-KVIxESLEETYxKLxExxxxx
>CON.235
-------xxCVFCxxxxxDxxxxIIxRGExxFVILNxYPYTSGHLMVVPYxHxxxLxxLxxExxxEMMxLxxxxxxVLRxxYxPxGINIGMNLGxAAGAGIAHLHLHVLPRWxGDTNT--x-VGET-RVLPExLxxTWxRLRxxxxxx
>CON.236
-----KxxGCFLCxYxxxDDDNLVxYRGRHCFxIMNKYPYNxGHVMVAPYRHVxxIxDLSxEELxECxxLxxxIxxALxKVLGxxDFxIGINIGRVAGAGIEHMHIHIIPRxPx------x-Ixxx-xxxxxxVxxxTxxLRExLxxx
>CON.237
-------xGCVFCxIxxNDEExxVLYRDExCFxVMNxYPYTPGHFMIIPHxHxDxxExLxxExWxHMSxxVQxGVKLLKExFGAxGVNIGMNLGxAAGAGIxHIHxHLVPRWxGDTNT--T-IGxT-RVYGxDFxxIYxKLKxxxxxx
>CON.238
--------------MSNEE---LIIMKNxxxxVxHNPxPFNNGHLIIAPxRHx-SINEIDxSxLxELMDLxKRFxxILxRVYNPHGFNVGIS-------LxPHVxIQVVPRWNGDVMT--LFYNVK-V-VPETVxESVSRIRxAVRE-
>CON.239
xx---xDVxCVFCAIAEG----xVLYNDxxNMVxMNIYPYNRGHLxVIPxKHYTDLNELxxEELKxLFxLVQRxxxLIREVIKPDGINVGINLGEAAGxSIxHLHIHIVPRFKxESME--T-TADT-RVIEExIxKFxEKID------
>CON.240
LV---PxxxCILCxIxxx----LxVxExExFIVSVNLYPYNPGHLIIFPRRHIxDxRELTxEExxELHExTxxxLxILDxxWxPxGFNIGYNMGxxSGGSIPHIHxHIVPRYPNExxD--I-LxGx-RIIIEDPERLxKxxx------
>CON.241
-----xxxxCxFCxxxxx----xIVxENxxAYAIxDxYPVTxxHxLIIPKRHVxDYFDLxxxExxxxxxLLxxxRxxIxxDxSVxGFNIGxNxGxxAGQTIFHxHIHLIPRRxGDVxN--P-RGGV-RGVIPxKxxY----------
>CON.242
------xxxCIFCxxxxx----xxIxENExAxAIYDKYPVNxGHxLIIPKRHxxSFFEATxEEIxxIxxLLxEVKExLDxKYxPDGYNIGVNVGxxAGQTIxHLHIHIIPRYxGDVEN--P-RGGI-RxIKxxLVxYxx---------
>CON.243
------xxxCxFCxxxxx----ExIxENDLxYAxYDxYPVSxGHMLIIPxRHVxxYFDxTxEERxAIxxLIxxxKxLLDxKYxPDGYNIGVNxGExAGQTVMHLHIHLIPRYxGDVxx--P-xGGV-RGVIPxKxxY----------

```
>CON.244
-x---xxxxCVFCQxxxx----xxVLENDLAxAFWDIHPVSKGHLLIIPKxHxxTFFDVxxxExxAMxxLIxxAKxLLDxxYHPDGYNIGINVGxxAGQTVMHCHIHLIPRYxGDxPx--x-xGxI-RxMLPxxxxxx----------
>CON.245
--------xCxFCxx--x----xxILENELxxAFFDRxPVxPGHLLIIPKxHRxDYFALTxxEKQAIxxLLQxGKxLLEExYQPDGYNIGxNCGxxAGQTIFHCHCHLIPRYxGDxPx--P-RGGV-RGVIPxRQxY-----------
>CON.246
-------xxCPFCEx--x----DxIFENELAxAFYDxxPVSEGHILIVPKRHxASYFELTKAERxAIDELLELCKxYLDExFHPDAYNIGINVGxAAGQTVFHCHVHLIPRYQGDVKN--P-TGGV-RGVIPEKQNYR----------
>CON.247
-----xxxxCPFCxxxxQ----xIVLENExCxFLQxxxxxLxGSGIIVPKxHRxTVFDLTxEEWxxTYxLLxEVKxYLDxxYxPDGYNIGWNxGxVGGQHIxHAHLHVIPRYxDEPxA----GKGI-RYxLKxxxNRRxxxx------
>CON.248
-----xxxxCRFCxxNxD----xPLxxNxxFYMLxSxDPxxPxAxMIVxxRHxESPFEMxxEEWADLGxMLxxAKxxLA-xxxPDGFTLGWNVGAVGGQHVFHAHLHVIxRFAGEPxx----GRGI-RxIxRxx--------------
>CON.249
-----xxxxSPFxxIPxx----EWIASNExAFAIxDxFPVTPGHTLVIxRRxVxxWWEATxxEQxxLMELVDxVRxxLDxxxxPDGYNVGFNAGxAAGQTVxHLHVHVIPRYxGDVxD--P-RGGV-RHVIPxRGNY-----------
>CON.250
--------MxxFxxIPxx----RxIYExExFFIIxDxFPVSPGHxLIISNxxKxDFFELSxEEKxxLxKLIxxAKxLIExExxPDGYNIGMNCGExAGQTVFHFHCHIIPRYxGDMEN--P-RGGV-RHxIPGKGxY-----------
>CON.251
-----xxxxCPFCxxxxD----GxLxxNxLAYVRxDxFPVSPGHLLIIPRRHxxDWFDLTxxEQxAIxELIxQAKxxLDxxYxPDGYNIGMNCGEAAGQTVMHMHCHLIPRYxGDxxD--P-RGGV-RxVIPEKxDYxxxx-------
>CON.252
-----xxxxCPFCxx--x----xxVLENxLAFAIYDRxPVTPGHxLIIPxRHVADWFDxTxxEQxAMLxLxxxxRxxLxxExxPDGFNIGVNCGxAAGQTIFHVHMHLIPRYxGDMxD--P-xGGV-RGVIPARQKY-----------
>CON.253
-----xxxxCxFCNPxxx----xLLTESATAYAIFDGYPVSKGHxLIIPKRHVSNYFELxFKEQSACWxMVNKVQxILxxEFxPDGFNIGININxxAGQxIxHxxIHIIPRYxGDxxN--x-KGGI-RxVIPxKxxx-----------
>CON.254
-----xxxxCPFCxLxxx----RIIxxNxxAxxIxDGFPVSPGHTLIIPRRHVASFFExTxxERxALxxLLxxAKxxLDxExxPDGYNIGINDGxAAGQTVPHLHIHLIPRYxGDxxD--P-RGGV-RWVIPEKAxYWxxx-------
>CON.255
-x---xxxDCxFCRxSxD----PIIxENRSFxAxFDxxPVNPGHAxIIPRRHVxSLFDLxExEQADYFDxIxGVRxxIExTxxPDAYTIGxNDGRAAGRSIDHLHIxLIPRYxGDVED--P-RGGI-RNVIPxRxxxxxxx-------
>CON.256
-----xxxxCPFCxxxxx----RVxFxxxxVxAIWDAFPVSPGHLLIVPRRHVxTWxDLxxxEQxAIWxAIxxAxxxIxxRxxPDGFNVGFNxGEAAGQTVxHFHLHVIPRxxGDMxD--P-RGGV-RHVIPxKGNY-----------
>CON.257
-----TExNCPYCNFDEY----DVIxKNDFGVILPEPNPLSKGHxVIIPLRHVSSFFDVTDKERKSLMSLLELARNELQLRHQPxGFHIGFNDGxVFGExSEHLHIHIIPRYExQxLK--x-RWGI-xxx------------------
>CON.258
-----xxxDCPFCTxSSE----xLLTESAxAxAVxxxxPxTxGHTIVVPKRHVASYFDLxxxEQRAxWxVVDRVKSxLNERxxPDGFxVGFxxGxAxDxxAGHAxIHLIPRxPxxSxx--x-PRx----V------------------
>CON.259
-------xxCxFCDxxxI----xxIYENxLxFAFxTNIPIxPGHxLVCPxRxVSxIDELTDGEIxALFDLxxxIxExMKKxFNADGFNYAWNEGxxAGQTVxHLHIHILPRKKGDxG-----IxES-RxxSPEQELxEVAEL------
>CON.260
-----xxxxCPFCxSxxx----xxxFxSExFxAIYNxSPILPGHSLIIPKRHVxSLFDLTxxELSxFMxFxRKVxxLLxKIFxTDAFDWxLQEGxAAGQSVxHLHMHIIPRKxGDLPE--P-GDWx-RxxLSExELxxIxxx------
>CON.261
-------MxCPFCxPxxE----xILYEDELIRILIDSYPANRGHLLVVPRRHVExWEELxEEEKxALLRGVELAMExLKExLxPDGFNVGINLGKAAGQTVPHLHIHVIPRWKGDCxx--P-RGGV-RKAVLDIEDENLSLK------
>CON.262
--------------PFLPYExDLVADISDTHVxLLNKFNVVDHHLLIVTRxFExQExxLxxxDFxALxxCLxE----------xDGL-xFYNGGxxAGASQPHKHLQLVP--------------------------------------
>CON.263
WAIHG-LxSxxxxNPFLPYExSMVxEAGExHVCLLNKFPVISPHLLICSKxFVPQxSxLSxADFxAWLxGFDx----------PDVF-GFYNCGPVAGASQPHRHMQLV---------------------------------------
>CON.264
-----QERKCFLCxxNLPxEQx-GIPFGxxYxILVNPFPIFPxHFTIPxxxHVPQxIxxRFxDMLDLARxLxD----------YT---IFYN-GPKCGASAPHxHFQAGNKGFMPIEx--D-xx------------------------
>CON.265
--xx---xxCPFCD-xExLxxI--IxxEGDxIWLKNKYPTLxxQVIIESxDxxGDISTYSxEHxxxLxxFALExWxEMxxSxKYKSVLxYKNxGPxSGGSLRHPHMQIVGLxxxD---------------------------------
>CON.266
-ExxxYSExCPFCRGNEHxxxxxxIxxDGQWKSVKNKYPIIDEHVIIDTYRHNGNFYNMSExEFxNMFxMYxNRYKxLxxxxGIxYINIFKNxLRDAGASLMHPHSQIx---------------------------------------
>CON.267
-HNKKFKEDCPFCxxxQHxTAxKIYDxQGSxKVVENKYPxVxFHVVIEGRDHSKxFYELSKEHIxEIIKAYIxVSKNIYExEFIKYVQVFKNNGRxAGASLQHAHSQII---------------------------------------
>CON.268
-DEx----xCPFCPGxESxTPPxRVGxxxxWRVFPNKFPIxDxHVIVESPDHxxxIxxLSxxEVxEIIKxYxDRFRxxKx---xxxVxIFxNQGxxSGASIxHPHSQLVxVP------------------------------------
```

>CON.269
xxIx-xxxxCPFCPExERxTPxD----GTRIVTFPNLYPFADxHVTVITRAHxV--ExFxxRQIxDALxGQI------xSLxxxGYxSINWNYLPSAGASIxHPHL--
>CON.270
---------CPFCxENxKMTPxKEIAExGRIVVFPNLFPYSKHNVVVFSxQHYVKLEEFTxxLIKDAFxAAQTYIQxVxxxxKxxYASINWNYLPxSGGSILHPHLHVI---------------------------------------
>CON.271
--VxMKxGVCPFCxExK-YHxKPIIxxxxHWxVTENxxPGxKHHFLxIxKxHIxxxxELTxxAxxELxxIFxxLxxKxx----IxGGxxIMRFGDxxGATVxHLHAHLIxGxxRxExx--ExLxxxLGYKxKK---------------
>CON.272
--VxIxxGxCPFCxE-K-WHxxPILKRxxDWFITRxxxPNTRxHFLIIGRxHxExFxELxxxDWxxITxLVRWAIKKFK----IRGGGLAMRFGDTDGATVxHLHxHLIVPNIKTGKA--xTVxFPIG--------------------
>CON.273
--YRMxAGICLFCPxRx-HEKQxVLWETxHWMVTPNEFPGTRLHLLLIPKEHVTDLLDLSxAARADFWEVLxxVRDRYG----LTHYGLGVRNGDCRGGTIRHLHVHVLxGxxExxxF--xPVRMKFSSxPxR---------------
>CON.274
--LEHKSxxCxFC---S-xESxQVVRExEHFxLVKNxFGIVxEHLMLIPKRHIxSLxELxxDExxEYVDIIxx----YE----xxGYSIYARAxxSxTKSVPHxHTHLIRIDGKxIKx--Lx----YxxxPHI--LxxR---------
>CON.275
-------MECPFCEI-DKx---RxIKxxKHCFVIFSNPRLMPGHLLVIPKRHVEKLxELxExExxDLFNxVIxYQEKIIKxI-AxGCDIRQNYRKQxxLKVDHLHIHLQPRDFEDEL-----YxKx-QxExELFExEIxKLLxx----
>CON.276
----LxDHxCPFCNx-Nxx---FxIxENxxxYIIxARAPYVEDHLLIIPKRHVIxLxNLxxxExxxIxxLIxxxxNxLHxK--Hxx--VxLxxRxSTGKTIxHLHFHLVPKxxIxx-------xxS-QxDKRKFxIxExNKLKxLx--
>CON.277
-----xxxxDxFCxE--EIIxRQLVYEGKHVxILYDYAPIGKLHFIIVPKxHRxxFxELTxDEYVEAxxLSxKLIxHYKEQ-GYSxxYIFHKxxRxAGQSVPHWHxHLIFTxxKxELx--VLxGSS-PLxDDELRRRVxxYRxELxx-
>CON.278
--xx-xxxxCPFCNx--xVLExQKFYEDDLVIGLxTYKPIxPGHCLIIPKRHVERFxELTDxEILRIGQVIKKVNxAAEKVFGTxSYILLQKNGxEVGQSVPHVHFHYIPRKKxx-Lx--FLxxKK-PLxxEEMxExVxKMKxAIEx-
>CON.279
P----xxxxCAFCxYLxGxRPFTILxRNxxVAVLVTREQRGxPHLLVIPVRHxPTILDLTDxExxALxxxVRxAAxAIDxAYxRPGIAVWQNNGV-xxQAIxHLHFHVAGTLPGGGTD--WGDP---ELxVxETDxIAxRIxPYxxx-
>CON.280
P----VRDPCDLCEGMAGRExWxVVDEGEHTLTVINPWQFEVGQCCVITRRHVATLLDLSDPECSAVMxAAKRVAEALVxAYQPLGILTFQNNGV-YGQETPHFHFHVVPRQP--GSD--WGIP---PVxAxQLAETVKRIRAHLPx-
>CON.281
-----xxxDCTFCDxE----ExKIxxRTKNFxVWLSIGQIVEGYCLIIPNDHYHCxGALPxxLxxEYLxLKExVRxILxExYG-xCI--FYEHGNVQPGLCYHAHLHAVPVxxDLxxx--xxEDGLFPIKLxxxxDxFxExGHYLYY-
>CON.282
------xxECxFCKILKxxxxNxILxETDNFIVFPxTGxFIEGYLLIIPKxHIxxFAELSxEELKELxxIINxxSEIxKKxYxxxxx--xFEHGxxSGxSIVHAHLHIIPxNIxxxxx--IxKxxxxxExIDxxNDxIxxYxxYLxY-
>CON.283
------xxxCVFCDRSQ--FEERLIxExExFYIIATLGQIxxGYVLLxPKRHVxCLGAMxxxExxxxxxxxxxVxxxLxxEYxxxxI--IFEHG---GQTIKHAHLHIIPxxxDLxxR--IKxDFxxIxxxxxLxxxYxRxxPYLF--
>CON.284
------xxxCxFCxExN--LxDRILYETENWxLVPALGSFIPGYLLIVSKxHFxSIxxCPEDIFNELExLLKxVxKLIxxxYxxxxI--xFEHGxKGGCCIDHxHLHILPFDGxIIx---ExxxxSxxxxFxDIxDLxIxNxxYLFYN
>CON.285
-----xxxxCEFCTELxDxIxxRIMxxxExFVVMPTIGxIxxxxLLVLPKRHYESxAQLxxNxIxELxxLLxxLxNKLxx-FG-xVV--xFEHGxxSxCGVYHAHIHIIPVPSxIDxx----xFLxxxENISxxxTFxLNxxQYLMx-
>CON.286
------MxDCQFCxEFAx-xKTRIITEADxWVLLPTIGCLTPGYxLFMPLEHLDAAADVxPADLxQVAAxLExMRxLIQxRYG-PxI--VAEHGExGASCCSHxHLHLIPVPDPDAIx--AEKVGGPGxRLxSLADLPAAExSYLYL-
>CON.287
------MPSCDLCxAIDPVxQPRVLAANxHAAAVPTIGAFVxGYLLVVPxxHVLSLGxLxxxERAGVDxLxRELAxRLxDVYxSPVL--GFEYGLPGxRRIxHGHLHLLPSTADLxGW--L---------------------------
>CON.288
--x--xIxxCRFCxxxxxxxxDRPLxESxxFxAIPSIGxLVEGWxLIVPKRHxLNFxxxxx--RxEFxxFASxVxxxVxxxYG-xVV--xFEHGSxTGCGVDHAHLHIVPxxxDLxxx--V-xDxDxxxxWxxIxxL--xxxEYLxx-
>CON.289
--x--xxRECTFCCEVExxxxDYVLYESDHFVVMPCIGALTDWYLLIVPKRHTLSVGWLDDAxxADLxxLIPQVTDxIRxRxGxExL--VFEHGDKGGACxDHTHIHVVPTxxDxxxF--LITMRPCxDWIxAARHxVERQRPYLxLx
>CON.290
--x--xxxDCVLCPPL---LPGGVLAxDxxFLLMPDLAPLADGHLLLVTRxHxQCAGxFGxxLWxRALRWRDRVAxLYRxAYGxDLL--LFEHGQGGGACIDHAHWHLLPGTxGVRAV--LExxGxPPAxHxALRARFRAGRSYLL--
>CON.291
------MxExDFCxELSGAPxSRIIxxTxxxTLIADMSPLVIGHLLLLPxxHYLSFAQVLxxHxxEVxxxLxxFxxLYRxTFG-Exx--ILEHGMxSxACITHAHLHxLPIDxDxxxL--MxxDGLxxxxLxSLxELAxxDSPYFL--
>CON.292
-x---xGDxCxFCQLMENPEQLxLVGETDNFYAWLExPPRAKGHTxVVPKEHxESVMDLSPSEYxExMxLVREVMEKAxEGLGADGVSVAMNIxEAGGQMMPHAYIQVFPRFEDDENA------------------------------

Figure S6. Alignment of cluster consensus sequences of the HIT domain proteins from completely sequenced genomes. The phylogenetic tree, constructed from the multiple alignment of 15,351 HIT sequences (Figure 6A) was split into subtrees at the average depth of 1.5 from the tree tips, producing 292 clusters of 4+ sequences. Consensus sequences were derived for each cluster from the corresponding subsets of the alignment (positions, less than 30% conserved denoted by “x”). Positions corresponding to active site histidine residues are highlighted in yellow. Consensus sequence for the $MccH^{Hmi}$ (CON.109), $HinT^{Eco}$ (CON.19), and $HinT^{Hmi}$ (CON.21) clades are shown in red font.
